# Spatial Treg niches and the therapeutic reprogramming of tumour tolerance in cancer

**DOI:** 10.3389/fcell.2026.1921036

**Published:** 2026-09-07

**Authors:** Jhommara Bautista, Miranda Di Capua Delgado, Juliana Viteri-Recalde, Melanie Benítez-Núñez, David Ramírez-Sánchez, Andrés López-Cortés

**Affiliations:** Cancer Research Group (CRG), Faculty of Medicine, Universidad de Las Américas, Quito, Ecuador

**Keywords:** biomarker-guided Treg targeting, immunotherapy resistance, regulatory T cells, spatial immune niches, tumour microenvironment

## Abstract

Regulatory T cells (Tregs) maintain immune homeostasis, but in cancer the same FOXP3-dependent programme can be co-opted to protect malignant tissue from immune elimination. This review critically synthesizes spatially resolved, single-cell and mechanistic evidence to determine when tumour-associated Tregs constitute active components of suppressive multicellular niches rather than merely correlates of immune exclusion. We integrate tumour-adapted regulatory states with anatomical positioning, neighbouring malignant and non-malignant cells, candidate suppressive mechanisms and upstream tumour-intrinsic, stromal and myeloid programmes. This framework distinguishes Treg-dominant suppressive niches from Treg-associated architectures in which regulatory-cell accumulation is secondary to other resistance mechanisms. We examine tumour nests, invasive margins, dendritic-cell and lymphoid aggregates, stromal and perivascular barriers, and hypoxic or metabolically constrained regions, assessing the strength of evidence linking each context to local immune restraint. We further consider how these niches emerge during tumour progression, change under therapeutic pressure, and persist, relocate or re-form during resistance. We evaluate selective depletion strategies targeting CCR8, CD25 and CTLA-4, together with functional reprogramming of TGF-β, adenosine, kynurenine, lactate, hypoxia and IL-2 pathways. Finally, we propose a tiered biomarker framework integrating Treg phenotype, transcriptional state, spatiotemporal topology, functional immune competence and longitudinal pharmacodynamic validation. The contribution of this Review is therefore not the niche concept itself, but its Treg-centred mechanistic and translational operationalization for identifying tumour-specific regulatory dependencies while preserving systemic self-tolerance.

## Introduction

Immune tolerance is indispensable for organismal survival, but in cancer it becomes a mechanism of tumour protection. Regulatory T cells (Tregs) evolved to restrain autoreactive, commensal-reactive and tissue-damaging immune responses, thereby preserving self-tolerance and limiting inflammatory injury. The identification of CD4^+^CD25^+^ suppressive T cells and FOXP3 as the lineage-defining regulator of this compartment established the FOXP3–Treg axis as a central mechanism of dominant peripheral tolerance (Sakaguchi et al., 1995; Fontenot et al., 2003; Hori et al., 2003). In malignant tissue, however, the same programme can suppress cytotoxic immunity, limit dendritic-cell priming, reinforce myeloid and stromal suppression, and convert antitumour inflammation into pathological tolerance (Shimizu et al., 1999; Nishikawa and Sakaguchi, 2010). Cancer does not invent immune tolerance; it misuses a physiological mechanism of tissue protection to preserve malignant tissue. Nevertheless, Tregs do not generate this state in isolation: tumour-intrinsic, myeloid, stromal and vascular programmes can initiate immune escape independently of Tregs or create the conditions that recruit and stabilize them. Tregs should therefore be interpreted as context-dependent components of tumour tolerance rather than as its universal upstream cause.

**FIGURE 1:**
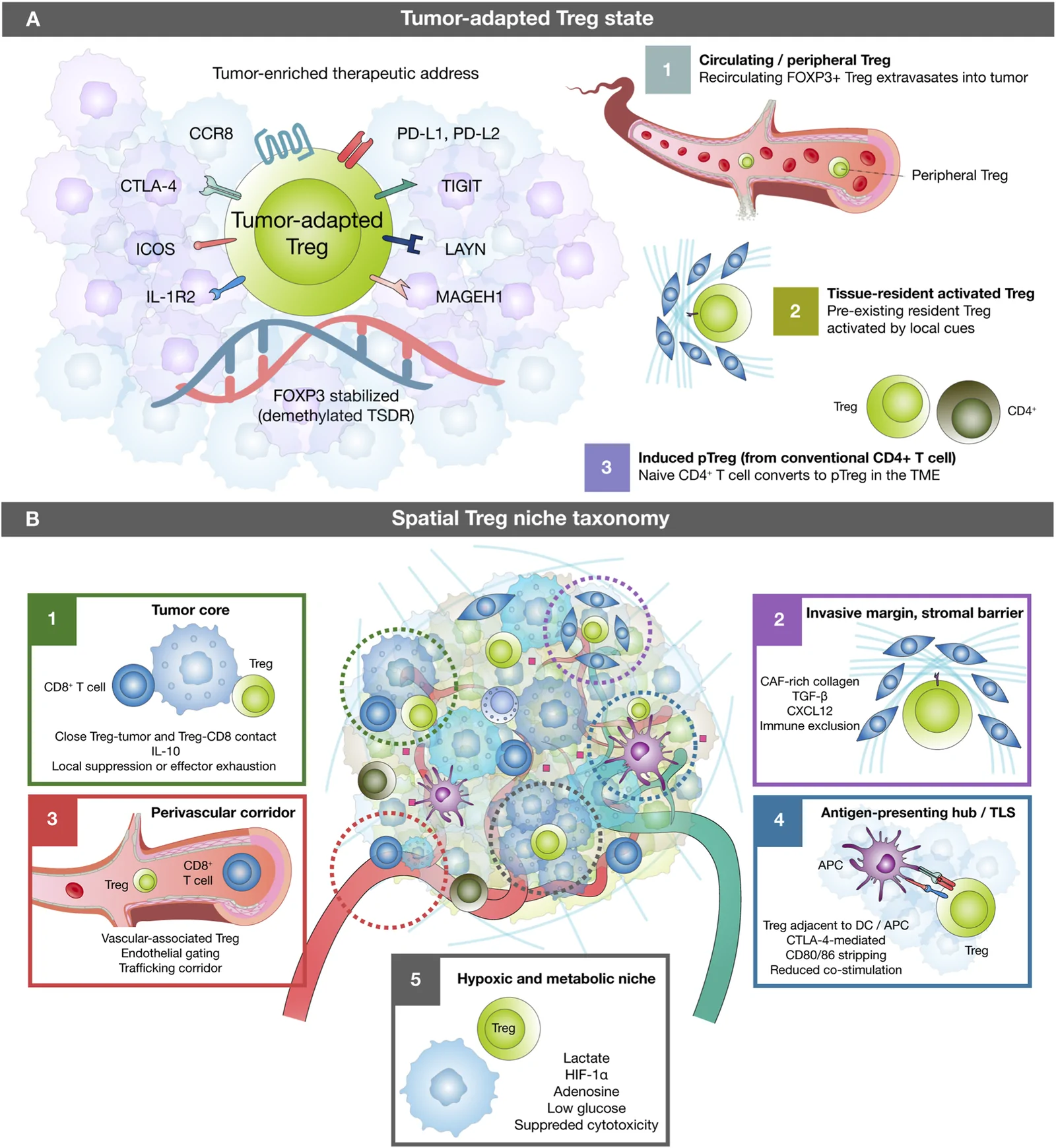
Tumour-adapted Treg states and spatial niche taxonomy. **(A)** Tumour-adapted Treg state and potential cellular origins. Tumour-associated Tregs retain a stable FOXP3-dependent regulatory identity, supported by demethylation of the Treg-specific demethylated region (TSDR), while acquiring a coordinated tumour-enriched programme comprising CCR8, CTLA-4, ICOS, IL-1R2, PD-L1, PD-L2, TIGIT, LAYN and MAGEH1. CCR8 is highlighted as a tumour-enriched and potentially therapeutically addressable surface marker. The intratumoral Treg compartment may arise through three non-mutually exclusive routes (A1) circulating or peripheral FOXP3^+^ Tregs extravasate into the tumour and undergo local adaptation; (A2) pre-existing tissue-resident Tregs become activated and reprogrammed by local malignant, stromal and inflammatory cues; and (A3) conventional naïve CD4^+^ T cells undergo peripheral conversion into induced pTregs within the tumour microenvironment. **(B)** Spatial taxonomy of tumour-associated Treg niches. The central tumour map illustrates the heterogeneous distribution of Tregs across five anatomically and functionally distinct microenvironments (B1) Tumour core: close Treg–tumour-cell and Treg–CD8^+^ T-cell contacts may support local suppression, IL-10-associated restraint and effector-cell exhaustion (B2) Invasive margin and stromal barrier: Tregs coexist with CAF-rich collagen, TGF-β and CXCL12 programmes that restrict immune-cell penetration and reinforce CD8^+^ T-cell exclusion (B3) Perivascular corridor: vascular-associated Tregs occupy trafficking routes in which endothelial gating and vascular dysfunction regulate immune access to tumour tissue (B4) Antigen-presenting hub or tertiary lymphoid structure: Tregs positioned adjacent to dendritic cells or other APCs can restrict local priming and restimulation through CTLA-4-dependent removal of CD80 and CD86 and consequent reduction of costimulation (B5) Hypoxic and metabolic niche: low glucose, lactate accumulation, HIF-1α-associated stress and adenosine-rich conditions impair cytotoxic lymphocytes while favouring the persistence or relative fitness of tumour-associated Tregs. Together, these niches demonstrate that the biological significance of Tregs depends on their cellular state, anatomical position and neighbouring suppressive circuitry rather than on abundance alone. APC, antigen-presenting cell; CAF, cancer-associated fibroblast; pTreg, peripherally induced regulatory T cell; TME, tumour microenvironment; Treg, regulatory T cell; TSDR, Treg-specific demethylated region; TLS, tertiary lymphoid structure.

Tumour-infiltrating Tregs are not merely circulating Tregs that have accumulated in cancer. Human transcriptomic and single-cell studies show that intratumoral Tregs acquire activated, suppressive and tumour-adapted programmes that distinguish them from peripheral-blood Tregs and, in several contexts, from Tregs in matched non-malignant tissues (De Simone et al., 2016; Plitas et al., 2016). These states retain the canonical FOXP3–CD25 regulatory core but show increased expression of *CTLA4, TIGIT, ICOS, CCR8, LAYN, MAGEH1* and *IL1R2*, indicating that tumour Tregs combine lineage identity with tissue residency and tumour-driven activation (Figure 1A). Their clinical meaning therefore cannot be inferred from FOXP3^+^ cell density alone. It depends on which regulatory state is present, where it is positioned, which cellular circuits surround it and whether the tumour is functionally dependent on that regulatory programme.

Spatial organization is already an established principle of tumour immunology. Spatial transcriptomics and highly multiplexed imaging have demonstrated that malignant, immune, stromal and vascular cells form regional compartments, cellular neighbourhoods and multicellular ecosystems with prognostic and therapeutic relevance (Keren et al., 2018; Schürch et al., 2020; Pelka et al., 2021; Elhanani et al., 2023). The contribution of this review does not lie in proposing spatial immune organization, introducing the niche concept or demonstrating that Tregs occupy anatomically distinct regions. Rather, it lies in integrating tumour-Treg cellular state, anatomical position, neighbouring-cell circuitry, upstream causality and therapeutic actionability within a single Treg-centred framework.

Equivalent numbers of Tregs may have different biological consequences depending on whether they occupy tumour nests, invasive margins, stromal barriers, perivascular corridors, hypoxic regions or tumour-associated lymphoid aggregates. Treg proximity to CD8^+^ T cells may indicate direct effector restraint; proximity to dendritic cells (DCs) may indicate control of antigen presentation, costimulation or local restimulation; and association with macrophages, fibroblasts, vessels or collagen-rich stroma may identify suppressive architectures that restrict immune access or function. However, spatial proximity alone does not demonstrate that Tregs mediate local suppression or causally organize the surrounding tissue. Tumour-intrinsic WNT–β-catenin signalling, for example, can impair dendritic-cell recruitment and drive T-cell exclusion independently of Tregs, whereas experimental Treg depletion can remodel myeloid, fibroblast and endothelial programmes in Treg-dependent settings (Spranger et al., 2015; Glasner et al., 2023). This Review therefore distinguishes Treg-associated neighbourhoods from mechanistically supported and functionally validated Treg niches. Accordingly, Treg biology should be mapped and mechanistically contextualized, not merely counted (Figure 1B).

Spatial organization is also temporally dynamic rather than fixed. Treg niches may be seeded during early tumour development, consolidated as malignant, stromal and vascular programmes evolve, and remodelled under therapeutic pressure. Immune-checkpoint blockade does not produce a uniform regulatory trajectory: the Treg compartment may remain numerically stable, acquire altered proliferative or suppressive states, expand within particular anatomical structures, or be replaced by compensatory myeloid and stromal circuits (Ribas et al., 2016; Kamada et al., 2019; Eschweiler et al., 2021; Glasner et al., 2023). Niche stability must therefore be considered across distinct dimensions, including persistence of Treg abundance, suppressive phenotype, spatial relationships and functional tumour dependence. This temporal perspective requires longitudinal comparison of pretreatment, early on-treatment, response and progression specimens rather than inference from isolated tissue snapshots.

This spatial, temporal, and causal perspective helps explain why immune activation does not always produce tumour rejection. Checkpoint blockade, cancer vaccines, radiotherapy and adoptive cell therapy require tumour-reactive lymphocytes to be primed, enter malignant tissue, receive local restimulation, survive metabolic and stromal stress, and execute cytotoxic function. Treg-dependent or Treg-reinforced niches can interrupt this sequence by limiting dendritic-cell costimulation, competing for cytokines, reinforcing suppressive myeloid and stromal states, and restraining effector cells within specific tissue regions. Response to programmed cell death protein 1 (PD-1) blockade is favoured by pre-existing CD8^+^ T cells positioned at the invasive margin or within tumour parenchyma, whereas immune-excluded tumours shaped by stromal TGF-β programmes can resist checkpoint therapy despite immune-cell presence (Tumeh et al., 2014; Mariathasan et al., 2018). The therapeutic significance of Tregs therefore depends not only on their abundance or position, but also on preserved antigen-presentation capacity, the presence of tumour-reactive effector cells and evidence that the suppressive circuit is functionally dependent on Treg activity. It may also depend on whether that circuit persists, adapts or is replaced following treatment. Resistance to immunotherapy should consequently be understood not only as failure to reinvigorate exhausted CD8^+^ T cells, but also as failure to establish and sustain a permissive immune architecture.

Therapeutically, this framework shifts the goal from global Treg depletion to tumour-restricted disruption of functionally relevant regulatory circuits. CCR8-targeted antibodies, Fc-optimized anti-CD25 strategies and Fc-dependent anti-CTLA-4 mechanisms illustrate that successful Treg targeting requires more than antigen expression: antibody format, Fcγ-receptor engagement, effector-cell availability and spatial access determine whether target binding results in intratumoral Treg depletion (Simpson et al., 2013; Arce Vargas et al., 2017; Van Damme et al., 2021). Complementary approaches may reprogramme the signals that stabilize Treg-containing niches, including TGF-β, adenosine, kynurenine, lactate, hypoxia and IL-2. Patient selection should therefore distinguish immune-inflamed tumours with an actionable Treg dependency from immune-excluded tumours driven predominantly by malignant, stromal or myeloid mechanisms. Longitudinal assessment should additionally determine whether the targeted regulatory circuit is disrupted, compensated for or re-established during treatment. The central challenge is to disrupt pathological tumour tolerance without compromising the systemic immune homeostasis maintained by Tregs.

Here, we critically synthesize evidence defining when and how tumour-associated Tregs participate in, amplify or dominate spatially and temporally organized immune tolerance. We examine tumour-Treg cellular states, anatomical distribution, temporal evolution and stability, multicellular circuitry, metabolic and epigenetic stabilization, immunotherapy resistance, therapeutic depletion and functional reprogramming. Throughout the Review, we distinguish spatial association from mechanistic support, functional dependency and therapeutic actionability, while considering how each of these properties may change during progression and treatment. We then translate this evidence into a biomarker and safety framework integrating Treg phenotype, transcriptional state, tissue topology, longitudinal niche remodelling and functional immune competence. The distinctive contribution of this Review is therefore not the spatial-niche concept itself, but its Treg-centred, causally discriminating and clinically operational spatiotemporal synthesis.

## The FOXP3–Treg axis as the foundation of immune tolerance

Immune tolerance is an actively enforced state. The modern concept of regulatory T cell biology emerged from the identification of a minor population of CD4^+^ T cells constitutively expressing the interleukin-2 receptor α-chain CD25, whose removal triggered systemic autoimmunity and whose reconstitution restored self-tolerance (Sakaguchi et al., 1995). This discovery established the principle of dominant peripheral tolerance: potentially pathogenic lymphocytes that escape central deletion are restrained in the periphery by a specialized suppressive lineage, now defined as CD4^+^CD25^+^FOXP3^+^ regulatory T cells.

FOXP3 converted this phenotypic population into a lineage-defining concept. Genetic studies of the scurfy mouse and human IPEX syndrome showed that disruption of FOXP3 causes catastrophic, early-onset immune dysregulation, establishing the FOXP3–Treg axis as indispensable for immune homeostasis (Bennett et al., 2001; Brunkow et al., 2001). Subsequent work showed that Foxp3 is enriched in CD4^+^CD25^+^ Tregs, required for their development and suppressive function, and capable in experimental systems of imposing regulatory properties on conventional T cells (Fontenot et al., 2003; Hori et al., 2003). Yet FOXP3 should be understood as the core organizer, rather than the sole determinant, of Treg identity, because forced Foxp3 expression can confer suppressive activity but does not by itself fully encode the magnitude, stability, or context dependence of the regulatory programme (Khattri et al., 2003). Stable suppressive function depends on FOXP3 operating within a broader regulatory network, and transient FOXP3 induction in activated human T cells does not necessarily produce a durable Treg programme (Gavin et al., 2006).

The physiological importance of this axis reflects a central paradox of adaptive immunity. A diverse antigen-receptor repertoire is essential for host defence, yet inevitably generates lymphocytes capable of recognizing self, commensal, dietary, and environmental antigens. FOXP3^+^ Tregs resolve this problem by limiting immune-mediated tissue damage without globally disabling protective immunity. In this setting, tolerance preserves tissue integrity, prevents autoimmunity, and maintains immune equilibrium at barrier and peripheral sites (Sakaguchi et al., 2008). Cancer co-opts this same logic. In healthy tissues, FOXP3^+^ Tregs restrain inappropriate immunity against self and innocuous antigens. In tumours, the same suppressive programme can restrain productive antitumour immunity, transforming a physiological mechanism of tissue protection into a pathological mechanism of tumour protection. The distinction lies in target and context: physiological tolerance protects the organism from immune-mediated damage, whereas tumour-associated tolerance protects malignant tissue from immune elimination (Sakaguchi et al., 2001; Nishikawa and Sakaguchi, 2010). Cancer does not invent immune tolerance; it hijacks the FOXP3–Treg programme to convert physiological immune restraint into pathological tumour protection.

## Tumour Tregs beyond tissue residency and chronic activation

Tumour-infiltrating regulatory T cells retain the canonical FOXP3–CD25 regulatory core but acquire transcriptional and functional programmes shaped by the malignant ecosystem (De Simone et al., 2016). However, comparison with peripheral-blood Tregs alone is insufficient to define a tumour-specific regulatory state, because adaptation to any non-lymphoid tissue is expected to distinguish resident Tregs from their circulating counterparts. The biologically relevant question is therefore which features of tumour Tregs exceed the programmes shared with normal tissue-resident, chronically stimulated, autoimmune and reparative Tregs.

Tissue residency and functional specialization are not unique to cancer. Visceral-adipose-tissue Tregs possess a distinctive TCR repertoire and acquire PPAR-γ-dependent metabolic programmes that control local inflammation and systemic metabolism (Feuerer et al., 2009; Cipolletta et al., 2012). Tregs accumulating in injured skeletal muscle express amphiregulin and promote tissue repair, whereas skin-resident Tregs occupy hair-follicle niches and regulate epithelial stem-cell differentiation (Burzyn et al., 2013; Ali et al., 2017). Single-cell profiling of skin, colon and their draining lymph nodes further demonstrated progressive trajectories of tissue adaptation that combine shared non-lymphoid-tissue programmes with organ-specific terminal states (Miragaia et al., 2019). Tissue residency, metabolic specialization, local proliferation and expression of activation or repair-associated molecules therefore cannot individually establish a uniquely tumoural Treg identity.

Persistent antigen exposure outside cancer can generate similarly activated and suppressive regulatory states. During chronic viral infection, highly suppressive Id3-low Tregs accumulate and express increased levels of immunomodulatory molecules, demonstrating that sustained activation and enhanced suppression are not restricted to malignancy (Rauch et al., 2017). Autoimmune tissues can likewise contain regulatory states that are absent from blood and are shaped by local stromal and myeloid signals. In rheumatoid and juvenile idiopathic arthritis, inflamed synovial tissues contain both highly suppressive CD25-high CXCR6-positive Tregs and dysfunctional CD25-low AREG-positive Tregs regulated by macrophage- and fibroblast-derived signals (Koh et al., 2026). These observations further show that tissue enrichment, chronic TCR stimulation, inhibitory-receptor expression and multicellular niche dependence are not, by themselves, tumour-specific properties.

Human tumour studies nevertheless identify a malignancy-conditioned layer superimposed on this shared tissue-adaptation programme. In colorectal cancer and non-small-cell lung cancer (NSCLC), tumour-infiltrating Tregs separate transcriptionally from matched normal-tissue and blood Tregs, display stronger suppressive activity and coordinately upregulate immune-regulatory molecules including *CTLA4, TIGIT, ICOS, IL1R2, CD274, PDCD1LG2, CCR8, LAYN* and *MAGEH1* (De Simone et al., 2016). Single-cell validation confirmed that these genes are co-expressed within *FOXP3* ^*+*^ *IL2RA* ^*+*^ tumour Tregs, supporting a genuine tumour-associated state rather than a bulk-tissue artefact. However, most of these molecules are also expressed by activated or tissue-adapted Tregs outside cancer. Their tumour-associated significance lies in their coordinated enrichment, cellular co-expression and relationship to enhanced suppressive activity relative to Tregs from matched non-malignant tissue, rather than in the presumed specificity of any individual marker.

Breast cancer adds an important refinement. Tumour-resident Tregs are not equivalent to activated peripheral blood Tregs; their global expression pattern partly resembles normal mammary tissue-resident Tregs. Tissue residency, therefore, contributes to their baseline identity. Malignant tissue, however, imposes an additional layer of specialization: breast tumour Tregs show stronger activation, proliferation, and suppressive features than their normal tissue counterparts, with enrichment of cytokine and chemokine receptor programmes. CCR8 emerges as one of the most differentially expressed receptors in tumour Tregs relative to peripheral blood Tregs, tumour conventional T cells, and most other immune populations (Plitas et al., 2016). Large-scale single-cell profiling of the breast tumour immune microenvironment further showed that tumour-infiltrating immune cells occupy an expanded phenotypic space relative to matched normal tissue, and that intratumoral T cells, including Tregs, distribute along continuous activation and differentiation trajectories shaped by TCR usage and environmental cues (Azizi et al., 2018). CCR8 should therefore be considered tumour enriched and potentially therapeutically addressable in selected malignancies, but not universally tumour exclusive.

Comparison with site-matched non-malignant inflammation provides the most stringent available test of malignancy specificity. In head and neck squamous cell carcinoma, the composition and phenotype of the immune infiltrate showed extensive congruence with non-malignant inflamed oral mucosa, demonstrating that many apparent tumour-associated immune features were instead general consequences of tissue inflammation. Nevertheless, HNSCC selectively enriched an ICOS^+^IL1R1^+^ Treg population showing recent TCR stimulation, clonal expansion and greater suppressive activity than IL1R1-negative tumour Tregs. Tumour-enriched interactions involving activated antigen-presenting cells, ICOSL–ICOS signalling and IL-1–IL1R1 signalling provided a local cellular context for this regulatory state (Mair et al., 2022). This study demonstrates that the malignancy-associated difference can reside in a composite receptor state and its supporting multicellular circuit rather than in a conventional activation marker considered in isolation.

Single-cell studies in additional cancers reinforce this state-based interpretation. In hepatocellular carcinoma, tumour Tregs form a *CTLA4*-high FOXP3^+^ population preferentially enriched in tumours rather than peripheral blood, expressing regulatory and activation-associated genes including *CTLA4*, *TIGIT*, *TNFRSF9*, *CCR8*, and *LAYN*, with evidence of clonal intratumoral enrichment (Zheng et al., 2017). The overlap between HCC tumour-Treg signatures and signatures reported in melanoma, breast, colorectal, and lung cancers suggests recurrent molecular modules across histologies, while allowing for tissue-specific variation. In NSCLC, activated tumour Tregs exhibit internal heterogeneity, including TNFRSF9-associated activation states and IL1R2-containing signatures associated with adverse outcomes (Guo et al., 2018). These findings indicate that tumours repeatedly enrich activated, clonally expanded and strongly suppressive regulatory states, but they do not establish that their individual molecular components are unique to cancer. What remains insufficiently resolved is whether reproducible composite Treg states or interaction circuits distinguish malignancy from site-matched chronic non-malignant inflammation across tumour types.

The malignancy-conditioned Treg delta should therefore be defined as the convergence of four properties: amplification of an activated suppressive programme beyond that observed in site-matched non-malignant tissue; selective or sustained antigen-driven clonal expansion; coupling to malignant, myeloid, stromal or vascular signals enriched within the tumour; and functional participation in tumour-protective immune restraint. No single marker currently defines this state across cancers. CCR8, IL1R2, LAYN, MAGEH1 and ICOS–IL1R1 identify context-dependent components of malignancy-conditioned programmes and should be interpreted as composite, tumour-type-specific signatures rather than universal identifiers.

Tumour Tregs should consequently be understood as specialized regulatory states generated at the intersection of lineage identity, tissue residency and malignancy-driven selection. They may arise through recruitment and local adaptation of peripheral Tregs, expansion of pre-existing tissue-resident populations or both (De Simone et al., 2016; Plitas et al., 2016; Panduro et al., 2016; Miragaia et al., 2019). Future studies should compare tumour Tregs not only with blood Tregs, but also with site-matched normal tissue, non-malignant inflamed tissue and, where feasible, Tregs from chronic infection, autoimmunity and reparative inflammation. A candidate programme should be considered malignancy enriched only when it exceeds this shared tissue-residency and chronic-activation background and is linked to tumour-enriched cellular interactions, functional suppression or clinically relevant tumour protection. Their principal distinguishing features are summarized in Table 1.

**TABLE 1:** Shared and distinguishing features of peripheral, tissue-resident, chronically stimulated and tumour-infiltrating regulatory T cells.

Treg compartment	Location and defining context	Principal features	Biological and therapeutic relevance
Peripheral Tregs	Blood and secondary lymphoid organs	Canonical CD4^+^FOXP3^+^CD25^high^ regulatory phenotype, including resting and activated states. Their transcriptional programme is less influenced by sustained tissue-specific signals than that of non-lymphoid Tregs	Maintain systemic self-tolerance and provide a circulating source from which tissue and tumour Tregs may be recruited. Non-selective depletion risks systemic autoimmunity and inflammatory toxicity
Non-malignant tissue-resident and reparative Tregs	Healthy or regenerating non-lymphoid tissues, including skin, colon, visceral adipose tissue and injured muscle	Acquire organ-adapted programmes controlling local tolerance, metabolism, epithelial integrity and tissue repair. These may include tissue retention, local proliferation, metabolic specialization and expression of reparative mediators such as amphiregulin	Establish the tissue-specific foundation on which tumour-associated states may develop. Their preservation is important because depletion may cause organ-specific inflammation, metabolic dysregulation or impaired tissue repair
Chronically stimulated or inflammation-associated Tregs	Chronic infection, autoimmunity and persistent non-malignant inflammation	Can display sustained TCR stimulation, clonal expansion, inhibitory and costimulatory receptors, enhanced suppressive activity and dependence on local myeloid or stromal signals. These features overlap substantially with many phenotypes attributed to tumour Tregs	Provide the essential comparator for determining whether a candidate tumour-Treg programme reflects malignancy or a generic response to persistent antigen and tissue inflammation. Their presence demonstrates that activation, clonality and tissue adaptation are not intrinsically tumour specific
Tumour-infiltrating Tregs	Malignant tissue and tumour-associated immune, stromal, vascular and metabolic niches	Retain the FOXP3–CD25 core and frequently show coordinated enrichment of activated suppressive programmes involving CTLA-4, TIGIT, ICOS, CCR8, IL-1R2, LAYN, MAGEH1 and related molecules. However, no individual marker is universally tumour specific. The malignancy-conditioned state is better defined by amplified suppression relative to site-matched non-malignant tissue, antigen-driven clonal selection, coupling to tumour-enriched multicellular signals and functional participation in tumour protection	Can restrain antitumour immunity and constitute candidates for selective depletion or functional reprogramming. Therapeutic relevance requires evidence of tumour enrichment, spatially plausible interactions, functional Treg dependency and sufficient residual effector competence

## Tumour-type heterogeneity of Treg states and niches

The FOXP3-dependent regulatory lineage provides a conserved foundation across cancers, but tumour-associated Tregs should not be regarded as a uniform pan-cancer population. Their phenotype, recruitment, anatomical positioning and functional importance are shaped by tissue of origin, oncogenic programmes, stromal composition, vascular structure and dominant inflammatory signals. Consequently, similar Treg abundance or phenotypic states may have different biological implications across tumour types, and no individual chemokine axis, spatial configuration or therapeutic vulnerability should be assumed to apply universally.

The prognostic significance of intratumoral FOXP3^+^ cells is similarly context dependent and should not be interpreted as uniformly adverse. Increased FOXP3^+^ T-cell infiltration has been associated with reduced survival in malignancies such as ovarian and breast cancer, consistent with suppression of productive antitumour immunity (Curiel et al., 2004; Bates et al., 2006). By contrast, high intratumoral FOXP3^+^ cell density has been associated with improved survival in several colorectal cancer cohorts (Salama et al., 2009). Pan-cancer analyses confirm that the direction and magnitude of this association vary according to tumour type, molecular subtype, disease stage and anatomical compartment (Shang et al., 2015). These apparently contradictory findings may reflect differences in the identity of the FOXP3^+^ population, including suppressive FOXP3^high^ Tregs and FOXP3^low^ non-suppressive CD4^+^ cells, as well as variation in tumour–stroma localization, CD8^+^ T-cell context, inflammatory or microbial exposure, mismatch-repair status and analytical thresholds (Saito et al., 2016). FOXP3^+^ cell density should therefore not be treated as a universal prognostic surrogate or as direct evidence that a tumour is functionally dependent on Treg-mediated suppression.

In melanoma, Treg accumulation can be linked to tumour-intrinsic oncogenic signalling. In an autochthonous BRAF^V600E^–PTEN-deficient melanoma model, oncogenic BRAF signalling regulated the expression of CCR4 ligands and promoted CCR4-dependent recruitment of tumour-activated Tregs from draining lymph nodes to emerging cutaneous lesions (Shabaneh et al., 2018). In established melanoma, the significance of this regulatory compartment also depends on the accompanying effector architecture: Tregs positioned within immune-inflamed lesions containing tumour-reactive CD8^+^ T cells and antigen-presenting cells may identify a potentially actionable regulatory context, whereas Treg accumulation in lesions lacking recoverable effector populations may have different therapeutic implications (Tumeh et al., 2014; Sade-Feldman et al., 2018). Melanoma niches may therefore combine oncogene-dependent Treg recruitment with local restraint of pre-existing antitumour immunity.

NSCLC displays a distinct pattern of regulatory-state heterogeneity. Single-cell profiling identified heterogeneous tumour Treg populations characterized by bimodal TNFRSF9 expression, with an activated Treg signature containing IL1R2 that was associated with poor prognosis in lung adenocarcinoma (Guo et al., 2018). Spatial analyses further indicate that tumour-cell–Treg proximity and enrichment of Tregs within tumour cores can carry prognostic information (Barua et al., 2018). In experimental lung adenocarcinoma, acute Treg depletion also induced early transcriptional remodelling of endothelial, fibroblast and myeloid populations, indicating that Treg-dependent regulation can extend beyond direct suppression of effector T cells to broader multicellular connectivity (Glasner et al., 2023).

Colorectal cancer illustrates why FOXP3^+^ infiltration cannot be interpreted uniformly. COREAD can contain both suppressive FOXP3^high^ Tregs and inflammatory FOXP3^low^ non-Treg CD4^+^ cells. The latter were associated with IL-12- and TGF-β-linked inflammatory programmes and with bacterial invasion of tumour tissue, particularly by *Fusobacterium nucleatum* (Saito et al., 2016). Variation in the balance between these populations may help explain why high total FOXP3^+^ cell density has been associated with favourable outcomes in some colorectal cancer cohorts, in contrast to the adverse associations observed in several other malignancies. Mismatch-repair status introduces an additional level of heterogeneity: MMR-deficient tumours are enriched for spatially coordinated immune hubs containing activated T cells together with malignant and myeloid cells expressing T-cell-attracting chemokines (Pelka et al., 2021). In COREAD, the niche framework must therefore distinguish suppressive Tregs from inflammatory FOXP3^+^ non-Treg populations and account for variation in microbiota-associated inflammation and mismatch-repair-dependent immune organization.

Breast cancer provides a different form of tissue adaptation. Breast tumour Tregs partly retain transcriptional features of normal mammary tissue-resident Tregs while acquiring enhanced activation and suppressive programmes, including increased CCR8 expression (Plitas et al., 2016). Their spatial organization is also relevant: CCR4^+^ Tregs recruited through CCL22 into lymphoid infiltrates surrounding primary breast tumours become highly activated and ICOS-high and are associated with adverse clinical outcome (Gobert et al., 2009). Breast tumours also range from immune-cold to mixed and spatially compartmentalized architectures, particularly within triple-negative disease, indicating that neither CCR8 expression nor overall Treg abundance defines a uniform breast-cancer niche (Keren et al., 2018).

Ovarian cancer illustrates chemokine- and hypoxia-dependent Treg recruitment. Tumour cells and tumour-associated macrophages can produce CCL22, promoting CCR4-dependent accumulation of suppressive Tregs within tumours and ascites (Curiel et al., 2004). In experimental ovarian tumours, hypoxia additionally induced CCL28 expression and the recruitment of CCR10^+^ Tregs, coupling immune tolerance to VEGF-associated angiogenic remodelling (Facciabene et al., 2011). These findings support a regulatory architecture involving malignant cells, macrophages, hypoxic territories and vascular programmes that differs from the immune-inflamed configuration observed in some melanomas and the lymphoid-infiltrate-associated organization described in breast cancer.

Pancreatic ductal adenocarcinoma represents an especially important boundary condition for the model. Cancer-cell-intrinsic FOXP3 can directly activate CCL5 and promote Treg recruitment in PDAC (Wang et al., 2017). However, in a genetically engineered mouse model of pancreatic carcinogenesis, experimental Treg depletion failed to relieve immunosuppression and instead accelerated tumour progression. Treg loss reduced TGF-β-dependent, tumour-restraining myofibroblasts and increased CCL3, CCL6 and CCL8 production, promoting CCR1-dependent myeloid recruitment and compensatory immune suppression (Zhang et al., 2020). These findings demonstrate that, at least in this experimental context, Tregs can suppress antitumour immunity while simultaneously sustaining stromal states that restrain more aggressive tumour-promoting circuits. Treg-directed intervention in PDAC must therefore consider the possibility of compensatory stromal and myeloid remodelling rather than assuming that depletion will necessarily restore productive immunity.

The proposed niche framework consequently does not imply that an equivalent Treg mechanism operates across all cancers. Rather, it provides a common set of questions that must be resolved within each tumour type: which Treg phenotype is present; which chemokine or retention signals position it; which anatomical compartment it occupies; which malignant and non-malignant populations sustain it; whether local suppression is functionally Treg-dependent; and which compensatory responses follow therapeutic disruption. Spatial biomarkers and therapeutic thresholds will therefore require tumour-type-specific validation rather than direct extrapolation from one malignancy to another.

## Tumour-intrinsic and stromal programmes upstream of Treg niches

Before causal importance is assigned to an intratumoral Treg population, the malignant-cell and stromal programmes that generate the surrounding immune architecture must be considered. In many tumours, Tregs are not the initiating source of immune exclusion but are recruited, maintained or functionally amplified within an environment established by tumour-intrinsic, fibroblast, vascular or myeloid mechanisms. The causal relationship can therefore proceed from malignant-cell state to immune exclusion and regulatory-cell accumulation, rather than from Tregs to the entire suppressive microenvironment.

Tumour-intrinsic WNT–β-catenin signalling provides a well-defined example of Treg-independent immune exclusion. In melanoma, activation of β-catenin suppresses tumour-cell CCL4 expression, prevents recruitment of BATF3-lineage CD103^+^ dendritic cells, impairs spontaneous CD8^+^ T-cell priming and produces resistance to anti-CTLA-4 and anti-PD-L1 therapy without requiring a primary Treg mechanism (Spranger et al., 2015). In this setting, the absence of productive dendritic-cell and effector-cell recruitment precedes any potential regulatory contribution. A Treg population detected within such a tumour should therefore not be assumed to organize immune exclusion unless its functional removal restores the missing dendritic-cell–T-cell axis.

Other malignant-cell alterations generate analogous forms of resistance. Tumour-cell PTEN loss activates PI3K–AKT signalling, reduces T-cell trafficking and susceptibility to T-cell-mediated killing and is associated with inferior outcome following PD-1 blockade in melanoma (Peng et al., 2016). Loss-of-function alterations affecting JAK1 or JAK2 can abolish tumour-cell responsiveness to IFN-γ, whereas B2M loss disrupts MHC class I antigen presentation and CD8^+^ T-cell recognition; both mechanisms have been identified in melanomas with acquired resistance to PD-1 blockade (Zaretsky et al., 2016). A malignant-cell transcriptional programme can also characterize spatially cold, T-cell-excluded regions and predict response to anti-PD-1 therapy, demonstrating that tumour-cell state itself can contribute to immune exclusion (Jerby-Arnon et al., 2018). Acquired resistance to MAPK-targeted therapy can similarly generate an immune-evasive tumour microenvironment and cross-resistance to immunotherapy in melanoma (Haas et al., 2021). NANOG-associated programmes provide a related connection between tumour-cell stemness and immune evasion by protecting stem-like malignant cells from antigen-specific cytotoxic T-cell killing (Noh et al., 2012). Tumour-intrinsic NF-κB-associated regulation can also restrict immune recruitment: constitutive expression of the NF-κB co-regulator IκBζ in melanoma suppresses CXCL9, CXCL10 and CCL5 expression, reduces NK- and CD8^+^ T-cell infiltration and promotes resistance to PD-1 blockade in experimental models (Kolb et al., 2025).

A particularly informative example directly connects an upstream stemness-associated malignant programme with a downstream Treg dependency. In NSCLC, tumour-cell-intrinsic SOX2 expression increases CCL2 production, promotes the recruitment of Tregs to peritumoural regions and induces resistance to immune-checkpoint blockade through Treg-dependent modulation of tumour-associated vasculature and exclusion of CD8^+^ T cells from the tumour core. Experimental depletion of GITR^+^ tumour Tregs restores CD8^+^ T-cell infiltration and, when combined with checkpoint blockade, improves tumour control (Torres-Mejia et al., 2025). This model demonstrates that an upstream tumour-cell programme and a downstream Treg-dependent mechanism are not mutually exclusive: malignant cells can initiate the suppressive architecture while recruited Tregs become necessary effectors of its spatial execution. Together with NANOG-associated immune resistance, this example establishes stemness-related malignant-cell states as potential upstream determinants of both immune exclusion and regulatory-cell dependency.

Stromal TGF-β represents a parallel upstream mechanism. In metastatic urothelial cancer, a TGF-β-response programme in cancer-associated fibroblasts was associated with retention of CD8^+^ T cells in collagen-rich peritumoural stroma and resistance to PD-L1 blockade; combined TGF-β and PD-L1 inhibition restored T-cell penetration in experimental models (Mariathasan et al., 2018). In genetically reconstituted models of metastatic colorectal cancer, cancer-associated fibroblasts constituted a major source of TGF-β, which suppressed TH1 and cytotoxic immune programmes and promoted T-cell exclusion. TGF-β inhibition restored immune infiltration and sensitized these tumours to PD-1–PD-L1 blockade (Tauriello et al., 2018). These studies demonstrate stromal exclusion mechanisms that do not require Tregs as their initiating cause, although Tregs may subsequently reinforce or stabilize the resulting suppressive architecture.

The relationship between WNT signalling and the regulatory compartment is more complex than a single linear WNT–FOXP3 pathway. WNT16B derived from ovarian fibroblasts can activate β-catenin in dendritic cells and induce a tolerogenic state that favours Treg differentiation (Shen et al., 2014). β-catenin stabilization can also enhance the survival and competitive persistence of established Tregs (Ding et al., 2008), whereas sustained Treg-intrinsic β-catenin activation can produce an altered IFN-γ/IL-10 programme and impair stable regulatory function under inflammatory conditions (Sumida et al., 2018). Because the latter findings were obtained predominantly outside tumour models, they establish the compartment-specific immunological potential of β-catenin rather than demonstrating a universal mechanism of tumour-Treg maintenance. WNT effects must therefore be assigned to the relevant cellular compartment: tumour-cell β-catenin can exclude dendritic cells and effector lymphocytes, stromal or dendritic-cell WNT signalling can favour regulatory differentiation, and Treg-intrinsic β-catenin can influence survival or functional state. These mechanisms may coexist but should not be collapsed into a universal direct induction of tumour Tregs.

This hierarchy yields three biologically distinct configurations. In a Treg-dependent tumour, selective regulatory-cell perturbation restores antigen presentation, vascular permissiveness or effector-cell function. In a Treg-reinforced tumour, an upstream malignant, stromal, vascular or myeloid programme initiates suppression, while Tregs amplify or stabilize it; successful treatment may therefore require combined targeting of both the initiating programme and the downstream regulatory compartment. In a merely Treg-associated tumour, regulatory cells accompany an immune-excluded state but are not required for its maintenance, making isolated Treg depletion unlikely to restore antitumour immunity. The central diagnostic question is consequently not whether Tregs are present, but whether they initiate, execute, reinforce or simply reflect the dominant suppressive programme. This distinction determines whether Treg depletion is likely to be sufficient, requires rational combination with upstream tumour- or stroma-directed treatment, or is biologically misplaced.

## The spatial architecture of Treg-mediated tumour tolerance

Tumour-associated tolerance is a spatial property of tissue. The suppressive impact of regulatory T cells cannot be inferred from abundance alone, because equivalent Treg numbers may have different biological consequences depending on whether they occupy the tumour core, invasive margin, stromal or perivascular corridors, or regions of tissue stress (Keren et al., 2018; Barua et al., 2018). The relevant question is where Tregs are positioned relative to malignant cells, CD8^+^ T cells, DCs, macrophages, fibroblasts, vessels, and anatomical boundaries.

This geographic view reflects a broader shift in tumour immunology. Broader tumour immune microenvironment (TIME) frameworks now distinguish immune-excluded, immune-inflamed and tertiary-lymphoid-structure-rich tumours, emphasizing that immune composition, functional state and cellular localization must be integrated to define clinically meaningful immune microenvironments and identify suppressive or activating micro-niches (Binnewies et al., 2018). Single-cell studies have shown that tumour-infiltrating T cells vary across cancer types, patients and tissue compartments, revealing distinct regulatory, exhausted, memory-like, cytotoxic and clonally expanded states in colorectal cancer and NSCLC (Zhang et al., 2018; Guo et al., 2018). However, dissociation-based profiling removes the spatial relationships that determine whether immune cells interact, exclude or bypass each other. Spatially resolved studies show that antitumour immunity is arranged into tissue regions, cellular neighbourhoods and multicellular hubs, rather than being distributed randomly across tumour tissue (Keren et al., 2018; Schürch et al., 2020). In human colorectal cancer, integrated single-cell transcriptomics and spatial profiling identified coordinated multicellular immune hubs, including a myeloid-rich inflammatory hub at the tumour–luminal interface and MMRd-enriched CXCR3-ligand^+^ foci containing activated T cells, illustrating how immune responses are organized as spatially restricted malignant–stromal–myeloid–lymphoid networks (Pelka et al., 2021). Treg-mediated tolerance should therefore be interpreted as a regional feature of tumour architecture, rather than only as a property of isolated FOXP3^+^ cells.

The tumour core and invasive margin represent distinct immunological territories. In the tumour core, Tregs may mark sites where effector cells are locally restrained or where cytotoxic infiltration has failed, consistent with spatial analyses showing that tumour-cell–Treg proximity and increased Treg infiltration into core tumour regions are associated with worse survival in NSCLC (Barua et al., 2018). At the invasive margin, Tregs may participate in boundary control, shaping the interface at which immune cells attempt to enter malignant tissue, a concept supported by landmark studies showing that immune-cell type, density and location in the tumour centre and invasive margin carry prognostic information, and that pre-existing CD8^+^ T cells at the invasive margin influence response to PD-1 blockade (Galon et al., 2006; Tumeh et al., 2014). A Treg-rich tumour core suggests intratumoral suppression, whereas Treg enrichment at the margin may indicate immune gating, stromal restriction, or failed penetration into tumour nests, particularly in immune-excluded tumours shaped by stromal TGFβ programmes (Mariathasan et al., 2018).

Stromal and perivascular regions add another layer of interpretation. In immune-excluded tumours, lymphocytes may accumulate in fibroblast-rich, stromal or vascular-adjacent regions without efficiently reaching malignant cells, consistent with studies showing that stromal TGFβ programmes, matrix-rich CAF barriers and desmoplastic perivascular compartments can prevent T-cell penetration into tumour nests (Mariathasan et al., 2018; Grout et al., 2022; Xiao et al., 2023). Multiplexed ion beam imaging in triple-negative breast cancer showed that tumours can be organized into cold, mixed and compartmentalized immune architectures, with immune-regulatory proteins such as PD-1, PD-L1 and IDO displaying cell-type- and location-specific expression patterns and tumour–immune border structures linked to survival (Keren et al., 2018). In this setting, Tregs may contribute to a regulatory geography in which immune cells are retained at stromal boundaries, tumour–immune borders or vascular entry routes, rather than suppressing CD8^+^ cells through direct contact inside tumour nests. In inflamed tumours, by contrast, Tregs and CD8^+^ T cells may coexist within the tumour bed, making short-range suppression more plausible. The distinction between inflamed and excluded tumours is therefore topological as well as quantitative (Tumeh et al., 2014; Mariathasan et al., 2018).

Cellular proximity is the operative variable within this topology. Treg proximity to CD8^+^ T cells suggests local effector restraint, whereas proximity to DCs implies regulation of antigen presentation, restimulation or immune licensing. Proximity to macrophages and fibroblasts points to multicellular suppressive neighbourhoods where regulatory immunity intersects with tissue repair, inflammation and matrix organization. Spatial separation can be equally informative, indicating compartmentalization rather than direct suppression (Barua et al., 2018; Keren et al., 2018).

In breast cancer, this spatial logic is supported by evidence that FOXP3^+^ Tregs located within lymphoid infiltrates surrounding the tumour, rather than within the tumour bed itself, predicted relapse and reduced survival. These lymphoid-infiltrate Tregs were functionally suppressive, locally activated and proliferative, enriched for ICOS, GITR and HLA-DR, and positioned near DC-LAMP^+^ DCs and CD8^+^ T cells, suggesting that suppressive Treg niches can form within tumour-associated lymphoid aggregates where antigen presentation and effector-cell restraint converge (Gobert et al., 2009). In colorectal cancer, FFPE-CODEX mapping of the invasive front showed that the immune tumour microenvironment can be decomposed into conserved cellular neighbourhoods, including T cell-enriched, macrophage-enriched, tumour-boundary, follicular and granulocyte-enriched regions, and that neighbourhood-specific immune states carry prognostic information (Schürch et al., 2020).

The regulatory DC–Treg circuit within such lymphoid environments should not, however, be presented as an entirely unresolved hypothesis. Tumour-migratory cDC2 s can transport tumour-derived antigens to tumour-draining lymph nodes and present them to conventional CD4^+^ T cells, yet fail to support productive antitumour CD4^+^ differentiation when restrained by Tregs. Experimental Treg depletion increased cDC2 migration and function, promoted ICOS^+^PD-1^low^ conventional CD4^+^ T-cell differentiation and generated cDC2-dependent antitumour protection. In human tumours, a higher cDC2-to-Treg ratio was similarly associated with protective CD4^+^ T-cell states and improved survival (Binnewies et al., 2019). This establishes a functional cDC2–Treg regulatory axis, although its anatomical implementation in human intratumoral and peritumoural lymphoid aggregates remains incompletely resolved.

The DC compartment is also regulated through acquisition of a mature regulatory state rather than solely through external suppression by Tregs. Single-cell analyses of human and mouse NSCLC identified mature DCs enriched in immunoregulatory molecules, termed mregDCs, which co-express maturation-associated genes such as CD40, CCR7 and IL12 B with regulatory molecules including PD-L1, PD-L2 and CD200. The mregDC programme can be acquired by both cDC1 s and cDC2 s following tumour-antigen uptake. AXL signalling promotes PD-L1 expression, whereas IL-4 restrains IFN-γ-dependent IL-12 production; experimental IL-4 blockade restored mregDC1 functionality, expanded tumour-infiltrating effector T cells and reduced tumour burden (Maier et al., 2020). Phenotypic DC maturation should therefore not be equated automatically with productive T-cell stimulation.

Spatial position constrains, but does not uniquely determine, the suppressive mechanisms available to Tregs. The canonical Treg repertoire includes regulation of antigen-presenting cells, cytokine deprivation, inhibitory cytokines, cytolysis and metabolic suppression (Vignali et al., 2008). Some niche–mechanism relationships are already supported by functional perturbation, whereas others remain spatially plausible but unproven in human tumours. The framework should therefore distinguish established regulatory circuits from anatomical assignments that remain inferential, rather than treating all proposed relationships as equivalent hypotheses.

Within immune-inflamed tumour nests, close Treg–effector proximity and high local cytokine demand favour short-range mechanisms. High CD25 expression can restrict locally available IL-2, whereas granzyme B- and perforin-dependent cytolysis provides a potential mechanism for eliminating nearby NK or CD8^+^ T cells (Pandiyan et al., 2007; Cao et al., 2007). These mechanisms are compatible with an inflamed compartment containing activated effector cells, but neither has yet been demonstrated to dominate specifically within human tumour cores.

In DC-rich or tertiary-lymphoid-structure-associated hubs, regulatory control comprises more than a single CTLA-4 mechanism. Tregs can remove CD80 and CD86 from antigen-presenting cells through CTLA-4-dependent trans-endocytosis, restricting CD28-mediated priming or local restimulation (Wing et al., 2008; Qureshi et al., 2011). In parallel, Tregs can restrain the migration and CD4^+^ T-cell-priming function of tumour-associated cDC2 s, whereas tumour-antigen-bearing cDC1 s and cDC2 s can acquire the mregDC programme and combine maturation with expression of inhibitory ligands (Binnewies et al., 2019; Maier et al., 2020). The mechanistically supported DC–Treg circuit therefore includes extrinsic Treg-mediated restraint of DC function and intrinsic regulatory reprogramming of antigen-bearing DCs. What remains unresolved is not whether these mechanisms exist, but where they dominate, whether they coexist within the same human lymphoid structures and which component constitutes the actionable dependency in an individual tumour.

At invasive margins, stromal barriers and perivascular corridors, suppression is more likely to involve multicellular immune gating than isolated Treg–effector contact. Fibroblast-derived TGF-β, extracellular-matrix organization, endothelial dysfunction and suppressive myeloid programmes can establish exclusion independently of Tregs, whereas regulatory cells may reinforce these barriers by limiting dendritic-cell activation or modifying vascular, stromal and myeloid states (Motz et al., 2014; Mariathasan et al., 2018; Glasner et al., 2023). In NSCLC, the tumour-cell SOX2–CCL2 axis provides a mechanistically informative example in which peritumoural Treg recruitment contributes to vascular restriction and CD8^+^ T-cell exclusion (Torres-Mejia et al., 2025). Stromal and perivascular niches should therefore be classified according to whether Tregs initiate, execute or reinforce the exclusion architecture.

Hypoxic and metabolically constrained regions favour a different combination of Treg recruitment, persistence and suppression. Hypoxia can recruit CCR10^+^ Tregs through tumour-cell-derived CCL28 and couple regulatory accumulation to VEGF-associated angiogenic remodelling (Facciabene et al., 2011). CD39 and CD73 can convert extracellular ATP into immunosuppressive adenosine, directly inhibiting nearby effector cells through adenosine-receptor signalling (Deaglio et al., 2007). Lactate uptake through MCT1 and reliance on oxidative metabolism can support the fitness and suppressive stability of intratumoral Tregs, while highly glycolytic tumours can increase PD-1 expression preferentially within the regulatory compartment (Watson et al., 2021; Kumagai et al., 2022). Importantly, lactate utilization and oxidative phosphorylation primarily sustain Treg persistence; unlike adenosine, they should not themselves be considered direct suppressive effector mechanisms.

Diffusible mediators such as IL-10 and IL-35 are difficult to assign to a single anatomical coordinate because they may originate from multiple malignant and non-malignant populations and act beyond immediate cell contacts. TGF-β requires a different interpretation because it is secreted predominantly as an inactive latent complex, making the site and cellular mechanism of activation at least as important as the source of the cytokine itself. Activated Tregs can present latent TGF-β1 through GARP/LRRC32 and activate it through integrin αvβ8, generating a short-range signal at the Treg surface (Tran et al., 2009; Worthington et al., 2015; Liénart et al., 2018). In experimental tumours, Treg-expressed αvβ8 locally activated tumour-derived latent TGF-β and suppressed neighbouring intratumoral lymphocytes (Lainé et al., 2021). Thus, TGF-β should not be classified solely as a broadly diffusible mediator: GARP–αvβ8-dependent activation provides a mechanism through which a tumour-Treg state can impose spatially restricted function. Functional attribution requires simultaneous identification of the latent-TGF-β source, the GARP- or αvβ8-expressing activating cell, the responding population and local SMAD signalling.

The niche–mechanism map proposed here is therefore an evidence-ranked framework rather than a catalogue of uniformly unresolved hypotheses. The cDC2–Treg axis, mregDC programme, CTLA-4-dependent costimulation editing and GARP–αvβ8-mediated TGF-β activation are mechanistically supported, although their relative contribution and spatial deployment across human tumours remain incompletely defined. Other assignments, including dominant IL-2 deprivation or cytolysis within particular tumour compartments, remain biologically plausible but require direct spatial perturbation. A niche should be considered mechanistically resolved only when its cellular participants, molecular interaction and local functional consequence have been demonstrated; therapeutic actionability additionally requires evidence that selective disruption restores antitumour immunity in the corresponding anatomical compartment.

The methodological implication is clear: Treg biology must be mapped rather than simply measured. Highly multiplexed protein imaging links cellular identity and functional state with spatial context, neighbourhood organization and clinically relevant tissue motifs (Bodenmiller, 2016). Spatial transcriptomics provides complementary broad molecular profiling while preserving tissue geography. Multiplexed imaging platforms, including imaging mass cytometry, MIBI and CODEX, can position Tregs relative to CD8^+^ T cells, DCs, macrophages, fibroblasts, tumour cells and vessels at single-cell resolution (Giesen et al., 2014; Goltsev et al., 2018). For DC–Treg niches, informative panels should distinguish cDC1, cDC2 and mregDC states and measure regulatory interactions rather than using pan-DC markers alone. Relevant features include cDC-lineage markers, CCR7 or LAMP3-associated maturation, CD40 and costimulatory ligands, PD-L1, PD-L2, CD200, Treg identity and the surrounding conventional CD4^+^ and CD8^+^ T-cell states. Integrated single-cell, spatial and perturbational approaches can then connect cellular state with anatomical position and establish whether an observed regulatory circuit is functional within intact tissue.

Current evidence supports tumour Tregs as context-dependent participants in spatially organized immune tolerance rather than as universal or apical organizers of the tumour microenvironment. Their anatomical position may provide information beyond overall abundance, but whether a spatial Treg pattern is causal, prognostic, treatment-associated or merely correlative depends on the surrounding malignant, stromal, vascular, myeloid and effector-cell architecture. In inflamed tumours, Tregs may directly restrain nearby antitumour lymphocytes, whereas in immune-excluded, stromal or hypoxic tumours they may reinforce architectures initiated predominantly by fibroblast, vascular, myeloid or tumour-intrinsic programmes. Spatial localization should therefore be used to prioritize candidate suppressive mechanisms, not to infer them conclusively. A Treg-rich region should therefore be classified as Treg-associated until perturbational evidence demonstrates local regulatory dependency, and as therapeutically actionable only when Treg state and positioning converge with functional dependence, residual effector competence and treatment-linked outcomes (Barua et al., 2018; Mariathasan et al., 2018; Glasner et al., 2023; Monkman et al., 2024; Cole et al., 2024).

## Temporal evolution and stability of Treg niches

Spatial maps provide only a snapshot of tumour immunity. Treg niches are dynamic structures that may emerge, consolidate, relocate or lose functional relevance as malignant cells evolve, stromal and vascular compartments are remodelled, metastatic sites are established and therapeutic pressures alter the immune microenvironment. For analytical purposes, their temporal evolution can be considered across four interconnected processes: initial Treg recruitment, stabilization of tumour-adapted regulatory states, treatment-induced remodelling and re-establishment or replacement during acquired resistance. These processes need not occur synchronously, and changes in Treg abundance, phenotype, spatial position and functional dependency may follow distinct trajectories. However, direct longitudinal evidence from serially sampled human tumours remains limited, and many proposed temporal trajectories are currently inferred from cross-sectional, pseudotemporal or preclinical studies.

During tumour development, Treg recruitment may begin through tumour-intrinsic or tissue-derived signals before a mature suppressive architecture is established. In melanoma, oncogenic BRAF signalling can induce CCR4-ligand production and promote Treg recruitment during early tumourigenesis (Shabaneh et al., 2018). Evidence from high-grade serous ovarian cancer further suggests that regulatory involvement differs between localized and metastatic disease. Pseudotemporal ordering of cross-sectional tumour samples identified changing tumour–immune interactions across an inferred progression trajectory, including MHC class II-associated recruitment of Tregs by keratin-expressing malignant cells in localized disease and greater Treg accumulation at metastatic sites (Egan et al., 2025). Although this approach does not constitute serial observation of the same lesion, it supports the hypothesis that Treg niches are progressively shaped by changing malignant, inflammatory and metastatic programmes.

Immune-checkpoint blockade can remodel regulatory compartments, but its effects are not uniform. In paired melanoma biopsies obtained before and during pembrolizumab treatment, intratumoral memory T cells expanded, whereas the overall frequency of cells with a regulatory T-cell phenotype did not change significantly, indicating that PD-1 blockade does not invariably alter total Treg abundance (Ribas et al., 2016). By contrast, in advanced gastric cancer, PD-1 blockade increased proliferating Ki-67^+^ effector Tregs in patients who developed hyperprogressive disease, whereas these cells decreased in patients without hyperprogression (Kamada et al., 2019). In syngeneic tumour models, anti-PD-1 treatment also increased intratumoral follicular regulatory T cells, which were concentrated within tertiary lymphoid structures and exhibited pronounced suppressive capacity and persistence (Eschweiler et al., 2021). Checkpoint blockade may therefore preserve overall Treg frequency while selectively expanding, activating or repositioning particular regulatory states, depending on the initial Treg phenotype, anatomical compartment and accompanying effector response.

The stability of a Treg niche should consequently not be defined by FOXP3^+^ cell persistence alone. We propose distinguishing four dimensions: numerical stability of the Treg population, phenotypic stability of its suppressive programme, topological stability of its cellular relationships and stability of tumour dependence on the regulatory circuit. A tumour may retain similar Treg numbers while their proliferative state, receptor expression, neighbouring cells or suppressive relevance changes. Conversely, perturbation of Tregs can rapidly remodel other niche components: acute Treg depletion in experimental lung adenocarcinoma induced early transcriptional changes in fibroblast, endothelial and myeloid populations before overt T-cell activation (Glasner et al., 2023). Treatment modalities other than checkpoint blockade can also alter niche topology. In paired NSCLC specimens obtained before and after neoadjuvant chemotherapy, treatment was associated with increased Treg density and infiltration and with altered spatial relationships between Tregs and CD8^+^ T cells (Cai et al., 2024). Thus, persistence of FOXP3^+^ cells does not necessarily imply stability of the broader regulatory niche.

Temporal validation should therefore become an essential component of Treg-niche analysis. Studies should incorporate baseline, early on-treatment, response and progression specimens whenever clinically feasible and integrate spatial imaging or transcriptomics with single-cell phenotyping, T-cell receptor tracking and functional measurements. Longitudinal analyses should determine whether candidate niches persist, relocate, undergo phenotypic reprogramming, recruit compensatory suppressive populations or re-emerge during acquired resistance. They should also distinguish true temporal evolution within the same lesion from differences between anatomical sites, patients or tumour subclones. A clinically actionable Treg niche should not be defined only by its pretreatment presence, but also by whether its early remodelling is associated with target engagement, restoration of effector immunity, therapeutic response or immune-related toxicity.

## Multicellular circuits that sustain Treg niches

**FIGURE 2:**
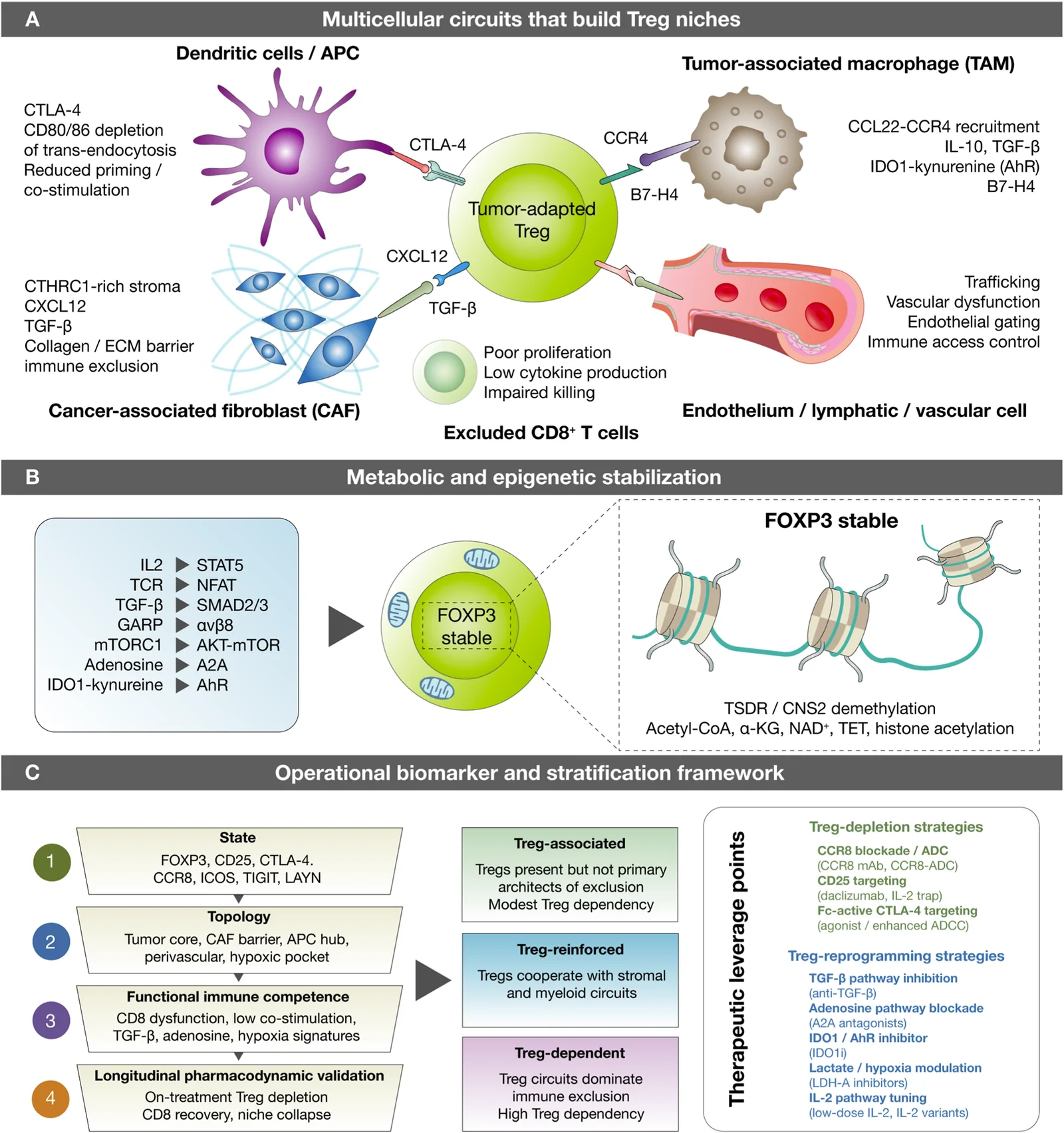
Multicellular circuits, metabolic–epigenetic stabilization and therapeutic stratification of tumour-associated Treg niches. **(A)** Multicellular circuits that build and sustain Treg niches. Tumour-adapted Tregs participate in reciprocal interactions with dendritic cells and other antigen-presenting cells, tumour-associated macrophages, cancer-associated fibroblasts and endothelial, lymphatic or vascular cells. Treg-expressed CTLA-4 can remove CD80 and CD86 from APCs through trans-endocytosis, reducing costimulation and productive T-cell priming. Tumour-associated macrophages can recruit CCR4^+^ Tregs through CCL22 and reinforce suppression through IL-10, TGF-β, IDO1–kynurenine–AhR signalling and B7-H4-associated programmes. CAF-rich territories provide CXCL12, TGF-β and collagen- or extracellular-matrix-dependent barriers that promote immune exclusion. Endothelial and lymphatic compartments regulate Treg and effector-cell trafficking through vascular dysfunction, endothelial gating and selective control of tumour access. The convergence of these circuits limits CD8^+^ T-cell proliferation, cytokine production and cytotoxic activity. **(B)** Metabolic and epigenetic stabilization of the tumour-Treg programme. Signals derived from IL-2–STAT5, TCR–NFAT, TGF-β–SMAD2/3, GARP–αvβ8, mTORC1–AKT–mTOR, adenosine–A2A and IDO1–kynurenine–AhR pathways converge to support regulatory-cell fitness and FOXP3 stability. These signals interact with mitochondrial metabolism and epigenetic mechanisms, including TSDR/CNS2 demethylation, acetyl-CoA availability, α-ketoglutarate, NAD^+^-dependent metabolism, TET activity and histone acetylation, to reinforce a durable suppressive state. The panel emphasizes that stable tumour-associated Treg identity results from the integration of extracellular signals, metabolic adaptation and epigenetic fixation rather than from FOXP3 expression alone. **(C)** Operational biomarker and therapeutic-stratification framework. Four complementary dimensions are integrated to determine whether a tumour contains an actionable regulatory dependency (C1) State, defined by markers such as FOXP3, CD25, CTLA-4, CCR8, ICOS, TIGIT and LAYN (C2) Topology, encompassing localization within the tumour core, CAF barrier, antigen-presenting-cell hub, perivascular region or hypoxic pocket; (C3) Functional immune competence, assessed through CD8^+^ T-cell dysfunction, reduced costimulation and TGF-β-, adenosine- or hypoxia-associated signatures; and (C4) Longitudinal pharmacodynamic validation, demonstrated by on-treatment Treg depletion or reprogramming, recovery of CD8^+^ T-cell activity and collapse or remodelling of the suppressive niche. Integration of these dimensions distinguishes Treg-associated tumours, in which Tregs are present but are not the principal architects of immune exclusion; Treg-reinforced tumours, in which Tregs cooperate with stromal, vascular or myeloid programmes; and Treg-dependent tumours, in which Treg-centred circuits constitute a dominant and potentially actionable mechanism of immune exclusion. Candidate therapeutic leverage points include Treg-depletion strategies directed against CCR8, CD25 or Fc-active CTLA-4, and Treg-reprogramming strategies targeting TGF-β, adenosine, IDO1–AhR, lactate, hypoxia or IL-2 pathways. A2A, adenosine A2A receptor; ADC, antibody–drug conjugate; ADCC, antibody-dependent cellular cytotoxicity; AhR, aryl hydrocarbon receptor; APC, antigen-presenting cell; CAF, cancer-associated fibroblast; CNS2, conserved non-coding sequence 2; DC, dendritic cell; ECM, extracellular matrix; IDO1, indoleamine 2,3-dioxygenase 1; mAb, monoclonal antibody; TAM, tumour-associated macrophage; TCR, T-cell receptor; TGF-β, transforming growth factor-β; Treg, regulatory T cell; TSDR, Treg-specific demethylated region.

Treg niches emerge from reciprocal circuits involving DCs, macrophages, fibroblasts, endothelial cells, and tumour cells. These accessory populations provide signals that recruit, activate, retain, and stabilize Tregs; in return, Tregs reshape the same cellular environment by limiting dendritic-cell costimulation, reinforcing suppressive myeloid programmes, cooperating with stromal barriers, and influencing vascular access. A Treg-rich region should therefore be viewed as a multicellular module in which antigen presentation, myeloid programming, stromal organization, endothelial gating and tumour-derived cues converge to restrain antitumour immunity (Wing et al., 2008; Qureshi et al., 2011; Curiel et al., 2004; Motz et al., 2014). This circuit-level view is directly supported by acute Treg-depletion studies in lung adenocarcinoma showing that fibroblasts, endothelial cells and myeloid populations undergo early transcriptional reprogramming before overt T-cell activation, revealing Treg-dependent accessory-cell connectivity and identifying VEGF- and CCR2-linked programmes as therapeutically exploitable niche responses (Glasner et al., 2023). These reciprocal interactions and their convergence on impaired CD8^+^ T-cell function are summarized in Figure 2A.

DCs are central to this circuitry because they determine whether tumour antigens are translated into immune activation or restraint. Productive antitumour priming requires DCs that capture antigen, provide costimulation, and license tumour-reactive T cells (Roberts et al., 2016; Broz et al., 2014). This requirement is illustrated by melanoma models showing that intratumoral CD103^+^ DCs uniquely transport intact tumour antigens to tumour-draining lymph nodes, prime tumour-specific CD8^+^ T cells, and are required for anti-PD-L1-mediated antitumour immunity. Therapeutic expansion and activation of CD103^+^ DC progenitors with systemic FLT3L followed by intratumoral poly (I:C)/poly-ICLC increased effector CD8^+^ T-cell infiltration, improved the CD8^+^ T-cell-to-Treg ratio and enhanced responses to PD-L1 and BRAF blockade (Salmon et al., 2016).

Tregs can interrupt this process through CTLA-4-dependent trans-endocytosis of CD80 and CD86 from antigen-presenting cells, leading to degradation of these costimulatory ligands, impaired CD28-mediated costimulation, and weakened T cell priming or restimulation (Qureshi et al., 2011). The *in vivo* importance of this pathway is supported by Treg-specific CTLA-4 deletion, which impairs Treg suppressive function, prevents Treg-mediated downregulation of CD80 and CD86 on DCs, causes systemic autoimmune disease, and unleashes potent tumour immunity, establishing CTLA-4 as a non-redundant effector mechanism of FOXP3^+^ Treg-mediated tolerance (Wing et al., 2008).

Regulatory control of DCs nevertheless extends beyond CTLA-4-mediated editing of costimulation. Tumour-migratory cDC2 s can transport and present tumour-derived antigens to conventional CD4^+^ T cells, but their capacity to generate protective antitumour CD4^+^ responses is restrained by Tregs. Experimental Treg depletion released this constraint, promoted cDC2-dependent differentiation of ICOS^+^PD-1^low^ conventional CD4^+^ T cells and improved tumour control. In human cancers, a higher cDC2-to-Treg ratio was similarly associated with protective CD4^+^ T-cell states and improved survival (Binnewies et al., 2019). The cDC2–Treg axis should therefore be considered a mechanistically established regulatory circuit rather than an unexplored consequence of spatial proximity.

DCs can also acquire an intrinsic regulatory state after tumour-antigen uptake. Single-cell analyses identified a conserved mature DC programme enriched in regulatory molecules, termed mregDC, which can arise from both cDC1 and cDC2 lineages. These cells combine maturation and migration-associated genes, including CD40 and CCR7, with immunoregulatory molecules such as PD-L1, PD-L2 and CD200 (Maier et al., 2020). AXL signalling contributes to PD-L1 induction, whereas IL-4 limits IFN-γ-dependent IL-12 production; experimental IL-4 blockade restored mregDC functionality and enhanced antitumour immunity. Phenotypic DC maturation should therefore not be equated automatically with productive T-cell stimulation.

The DC–Treg niche consequently comprises at least two complementary regulatory layers: extrinsic Treg-mediated restriction of DC costimulation and cDC2-dependent CD4^+^ T-cell priming, and intrinsic acquisition of the mregDC programme by tumour-antigen-bearing DCs. What remains unresolved is not whether these mechanisms exist, but how they are spatially distributed, whether they coexist within the same human lymphoid structures and which component constitutes the dominant therapeutic dependency in an individual tumour.

Tumour-associated macrophages provide a second circuit of niche maintenance. TAMs can recruit Tregs through chemokine axes such as CCL22–CCR4 and related CCL/CCR pathways, while producing or responding to immunoregulatory cytokines that favour suppressive myeloid states. In gastric cancer, CCL17^+^ and CCL22^+^ CD14^+^ cells are enriched within tumour tissue, correlate with FOXP3^+^ Treg accumulation among tumour-infiltrating lymphocytes, and promote CD4^+^CD25^+^ Treg migration *in vitro*, indicating that myeloid chemokine programmes can recruit Tregs early during tumour development (Mizukami et al., 2008). IL-10 and TGF-β connect macrophage polarization, Treg function, and impaired effector T cell activation. In turn, Tregs can reinforce suppressive myeloid programmes, creating a feed-forward loop in which macrophages attract or support Tregs, Tregs stabilize myeloid suppression, and both populations reduce local antitumour immunity. This myeloid arm of Treg niches is further supported by ovarian cancer data identifying B7-H4^+^ tumour-associated macrophages as a suppressive stromal population that inhibits tumour-antigen-specific T cell proliferation, cytokine production and cytotoxicity. Their B7-H4 expression was induced by tumour-environmental IL-6 and IL-10, exceeded the prevalence of CD4^+^CD25^+^ Tregs in ascites, and blockade of macrophage B7-H4 restored T cell activity and reduced tumour growth *in vivo*, supporting a broader suppressive circuit in which Tregs and regulatory myeloid cells cooperate to disable antitumour immunity (Kryczek et al., 2006).

Cancer-associated fibroblasts add the stromal scaffold on which many Treg niches are organized. CAF-rich regions remodel extracellular matrix, alter tissue stiffness, create stromal barriers, and shape chemokine gradients that influence immune-cell positioning. Single-cell and *in situ* analyses in head and neck cancer have shown that CAFs localize near invasive tumour-cell states at the leading edge and provide stromal ligand programmes, including TGF-β-related, FGF, and CXCL12-associated signals, illustrating how fibroblast-rich territories can organize malignant-cell invasion and local immune architecture (Puram et al., 2017). TGF-β links fibroblast activation, matrix remodelling and regulatory immune programmes, while chemokine fields involving CCL2, CCL17 and CCL22 can contribute to the recruitment or retention of suppressive immune cells, depending on the tumour context and cellular source. The Treg–CAF niche is therefore both architectural and immunological.

CAF–macrophage cooperation can further stabilize suppressive stromal territories. Pan-cancer single-cell and spatial analyses have identified CTHRC1^+^ extracellular-matrix-related CAFs as a recurrent stromal population enriched across multiple tumour types and preferentially positioned at the leading edge between malignant and non-malignant regions, where they may restrict immune infiltration. These CTHRC1^+^ CAFs colocalize with SLPI^+^ profibrotic macrophages to form spatial ecotypes characterized by extracellular-matrix remodelling, fibrotic programmes and immune-regulatory interactions, providing a pan-cancer framework for how stromal–myeloid territories can shape local tumour immunity (Han et al., 2025).

Endothelial and lymphatic endothelial cells regulate whether immune cells enter, remain within, or exit tumour tissue. These vascular compartments influence immune trafficking, stromal retention, and immune exclusion. Tregs can modulate endothelial and lymphatic programmes, while vascular-associated signals shape the routes through which Tregs and effector cells access tumour regions. VEGF-linked programmes are especially relevant because they connect angiogenesis, vascular dysfunction, and immune restraint. Treg–endothelial circuits may therefore determine whether tumour vasculature functions as a permissive gateway or as a selective barrier favouring regulatory dominance (Motz et al., 2014; Piao et al., 2022; Glasner et al., 2023). Neural and neuroendocrine inputs may add a further host-level layer to this vascular and multicellular control. Autonomic and neuroendocrine circuits can influence innate immune thresholds, dendritic-cell priming, endothelial and lymphatic trafficking, metabolic fitness, circadian immune timing, and durable immunosuppressive bias within the tumour microenvironment. These signals should not be viewed as separate from local tumour immunity, but as systemic regulators that may reinforce or constrain the multicellular circuits through which Tregs, myeloid cells, stromal cells and vascular compartments shape pathological tolerance (Bautista and López-Cortés, 2026b). Neurodegenerative remodelling may further amplify this host-level control. Degenerating neurons can disrupt DNA repair, mitochondrial function, immune regulation and barrier integrity, while promoting microbial dysbiosis and tumour-permissive inflammatory niches, particularly along enteric neuro–immune–microbiota axes relevant to colorectal and pancreatic cancer (Bautista et al., 2025).

Tumour cells provide many of the initiating cues that seed these circuits. Malignant cells can produce chemokines that attract Tregs, cytokines that condition accessory immune cells, and metabolites that influence regulatory stability. In pancreatic ductal adenocarcinoma, cancer-cell-intrinsic FOXP3 directly transactivates CCL5, thereby recruiting FOXP3^+^ Tregs into the tumour microenvironment and linking malignant-cell transcriptional state with regulatory immune accumulation and poor prognosis (Wang et al., 2017). In ovarian cancer, tumour cells can also induce CD8^+^ T cells with regulatory features, including increased FOXP3 and CTLA-4 expression, reduced CD28 expression, and suppressive activity partly mediated by TGF-β1 and IFN-γ, illustrating how malignant cells can directly promote regulatory immune states (Zhang et al., 2015). Tumour hypoxia illustrates a parallel principle: stressed malignant cells can induce Treg-attracting chemokines such as CCL28, linking tumour-cell adaptation, Treg recruitment, immune tolerance, and angiogenic remodelling. In ovarian cancer models, hypoxia induced tumour-cell CCL28 expression through HIF1α, promoting the recruitment of CCR10^+^ FOXP3^+^ Tregs that dampened effector T cell function, increased IL-10 and VEGFA-rich angiogenic remodelling, and accelerated tumour growth; depletion of CCR10^+^ or CD25^+^ cells reduced these tumour-promoting effects (Facciabene et al., 2011). Tumour- or myeloid-derived CCL22 can similarly attract CCR4^+^ Tregs and promote their accumulation in malignant tissue. This mechanism was established in human ovarian carcinoma, where tumour cells and tumour-associated macrophages produced CCL22, CCR4^+^ Tregs preferentially accumulated in tumour tissue and ascites rather than draining lymph nodes, suppressed tumour-antigen-specific T cell immunity, and were associated with reduced patient survival (Curiel et al., 2004).

This circuit-based framework positions Tregs as context-dependent effectors and amplifiers of pathological tolerance rather than its universal upstream organizers. In immune-inflamed tumours, Tregs may directly control DC costimulation, cDC2-mediated CD4^+^ T-cell priming and local effector function. However, DC regulation may also arise through the intrinsic mregDC programme, meaning that Treg-mediated restraint and DC-autonomous regulatory maturation can coexist without being mechanistically interchangeable. In other tumours, malignant, fibroblast, vascular or myeloid programmes establish immune exclusion before Tregs accumulate, with regulatory cells subsequently reinforcing or stabilizing the suppressive state (Spranger et al., 2015; Motz et al., 2014; Mariathasan et al., 2018). The causal direction must therefore be resolved experimentally. Multicellular association demonstrates participation, whereas selective perturbation is required to determine whether Tregs, cDC2 restraint, mregDC programming or another accessory-cell pathway constitutes the dominant functional dependency. The remaining frontier is the spatial and therapeutic resolution of these established circuits across human tumours, not the existence of the circuits themselves.

## Metabolic and epigenetic stabilization of suppressive Treg programs

Tumour-infiltrating Tregs persist in microenvironments that are metabolically hostile to many effector lymphocytes. Solid tumours commonly combine low glucose availability, lactate accumulation, hypoxic stress, amino-acid depletion, lipid-rich signals, mitochondrial stress, and limiting growth-factor availability. These conditions weaken glycolysis-dependent effector and cytotoxic T cells, yet can favour Tregs because the regulatory lineage is comparatively adapted to nutrient restriction, oxidative metabolism, lipid utilization and suppressive function under bioenergetic pressure (Chang et al., 2015; Ho et al., 2015; Angelin et al., 2017). The tumour microenvironment can therefore select for regulatory cells that survive where antitumour lymphocytes become functionally constrained. This fate-shaping role of metabolic signalling is supported by mTOR-deficiency studies showing that T cells can undergo initial activation and IL-2 production but fail to differentiate into Th1, Th2 or Th17 effector lineages; instead, under normally activating conditions, they acquire functional Foxp3^+^ regulatory phenotypes, indicating that mTOR signalling acts as a central metabolic checkpoint separating effector commitment from regulatory T cell differentiation (Delgoffe et al., 2009).

A central feature of this metabolic licensing is the low-glucose, high-lactate state of many tumours. Effector T cells require glycolysis for proliferation, cytokine production, and cytotoxic activity, making them vulnerable when glucose is scarce and lactate is abundant. FOXP3 can reprogramme T cell metabolism by suppressing MYC-driven glycolysis, increasing oxidative phosphorylation, and raising the NAD ratio, thereby allowing Tregs to retain proliferative and suppressive capacity in low-glucose, high-lactate environments that impair effector T cell fitness (Angelin et al., 2017). Lactate should therefore be viewed not only as a metabolic by-product of tumour glycolysis, but also as a selective pressure that tilts the immune balance toward regulatory dominance.

Hypoxia adds complexity. It is not intrinsically synonymous with Treg stability: HIF-1α can directly tilt the TH17/Treg balance by promoting RORγt-dependent TH17 differentiation while binding FOXP3 and targeting it for proteasomal degradation, thereby reducing Treg differentiation in inflammatory settings (Dang et al., 2011). In tumours, however, hypoxia rarely acts alone. It coexists with lactate accumulation, adenosine generation, altered nutrient availability, and inflammatory stress. These combined pressures create a metabolic ecology in which effector cells are disadvantaged while Tregs remain comparatively resilient. The key concept is that tumour hypoxia contributes to a broader suppressive metabolic landscape.

Extracellular nucleotide metabolism reinforces this landscape. Tregs frequently express CD39 and CD73, which convert extracellular ATP into adenosine, establishing an ectonucleotidase-dependent suppressive pathway in which ATP hydrolysis and adenosine generation attenuate effector immunity (Borsellino et al., 2007; Deaglio et al., 2007; Mandapathil et al., 2010). This is immunologically consequential because ATP can function as an inflammatory danger signal, whereas adenosine suppresses effector T cell activation and promotes immune restraint through adenosine receptor signalling, including A2A receptor-dependent inhibition of antitumour T cells (Ohta et al., 2006). A parallel logic applies to the IDO1–kynurenine–AhR axis. IDO1-mediated tryptophan catabolism can limit effector T cell responses while generating kynurenine-derived signals that support regulatory immune programmes through AhR-dependent pathways (Sharma et al., 2007; Mezrich et al., 2010). Together, adenosine and kynurenine convert metabolic stress into immunological suppression, a principle reinforced by tumour models showing that IDO/TDO–kynurenine–AhR activity sustains suppressive Treg–macrophage circuits and resistance to checkpoint blockade (Campesato et al., 2020).

A further layer of metabolic regulation may arise from microbiome-derived signals. Gut-derived and tumour-resident microbial communities can generate metabolites and microbial-associated molecular patterns, including short-chain fatty acids, bile acids, tryptophan-derived metabolites and lipopolysaccharide, that shape immune differentiation, antigen presentation, macrophage polarization and metabolic adaptation within the tumour microenvironment. These effects are context dependent and may promote either antitumour immunity or regulatory immune suppression according to metabolite concentration, receptor engagement, microbial localization and the pre-existing inflammatory or metabolic state of the tissue (Bautista et al., 2026a). This concept is particularly relevant in colorectal cancer, where dysbiotic microbial communities and biofilm-structured ecosystems can reprogramme immune, metabolic, neural and endocrine networks within the tumour microenvironment. Such host–microbiome axes may influence whether regulatory niches remain inflammatory, metabolically suppressive or therapeutically permissive, reinforcing the idea that tumour-associated tolerance can be shaped by both local tissue architecture and microbial ecology (Bautista et al., 2026c).

Treg persistence also depends on lipid handling, mitochondrial adaptation, and cytokine economy. Compared with glycolysis-dependent effector cells, Tregs can rely more heavily on oxidative metabolism, fatty-acid utilization, and mitochondrial fitness during nutrient limitation (Michalek et al., 2011; Weinberg et al., 2019). This does not mean that Treg metabolism is fixed; activated and tissue-adapted Tregs can increase glycolysis, lipid biosynthesis or mitochondrial metabolism when required, with mTORC1 linking TCR and IL-2 signals to lipogenic programmes, suppressive molecules and functional fitness (Zeng et al., 2013; Chapman et al., 2018). Their advantage lies in metabolic flexibility. IL-2 consumption belongs to the same resource economy: high CD25 expression allows Tregs to capture limiting IL-2, supporting STAT5-dependent survival and FOXP3 maintenance while restricting IL-2 availability for competing effector cells (Setoguchi et al., 2005; Pandiyan et al., 2007).

Suppression must also be epigenetically stabilized. Stable Treg identity depends on sustained FOXP3 expression embedded within a Treg-specific chromatin landscape. This distinction is especially important in humans, where conventional T cells can transiently express FOXP3 after activation without acquiring a stable suppressive lineage. True Treg commitment requires epigenetic fixation, including demethylation of the FOXP3 TSDR/CNS2 region and broader Treg-specific CpG hypomethylation patterns that support lineage stability, suppressive function, and resistance to effector-like conversion. This principle was established by studies showing that natural Foxp3^+^CD25^+^CD4^+^ Tregs display complete demethylation and active histone marks at an evolutionarily conserved regulatory region of the foxp3 locus, whereas TGF-β-induced Foxp3^+^ Tregs show only incomplete demethylation and lose both Foxp3 expression and suppressive activity after restimulation without TGF-β, demonstrating that FOXP3 expression requires epigenetic fixation to become a stable regulatory lineage programme (Josefowicz et al., 2012; Floess et al., 2007). This lineage-level stability is further supported by comparative human–mouse epigenomic analyses showing that Treg identity is specified by a small conserved set of lineage-specific regulatory elements associated with core genes such as *FOXP3, IL2RA, CTLA4, IKZF2, CCR8* and *LRRC32*; genetic variation within Treg-specific enhancers can alter epigenetic activity and is enriched for autoimmune disease-associated polymorphisms, underscoring how non-coding regulatory architecture shapes Treg function and immune tolerance (Arvey et al., 2015).

LRRC32 is functionally important because it encodes GARP, a surface receptor that anchors latent TGF-β1 on activated Tregs. Treg-expressed integrin αvβ8 can activate this GARP-bound latent complex, generating biologically active TGF-β in close proximity to responding cells (Tran et al., 2009; Worthington et al., 2015; Liénart et al., 2018). This mechanism directly connects a stabilized tumour-Treg state with spatial function: the source of latent TGF-β may be a malignant or stromal cell, whereas GARP^+^αvβ8^+^ Tregs determine where the cytokine becomes active. In melanoma and breast-cancer models, Treg-specific Itgb8 deletion reduced intratumoral TGF-β signalling, enhanced CD8^+^ T-cell activity and improved tumour control (Lainé et al., 2021). GARP, αvβ8 and total TGF-β should therefore not be treated as interchangeable markers; functional attribution requires identification of the latent-cytokine source, activating cell, responding population and local SMAD signalling.

The consequences of localized TGF-β activation remain context dependent. Integrin-β8-expressing type 1 Tregs can increase local TGF-β bioavailability and promote CD8^+^ TRM formation in infection models, linking the same activation machinery to tissue residency rather than only to suppression (Ferreira et al., 2020). Conversely, GARP deletion has not impaired tumour progression in every experimental model, indicating that alternative activating cells or suppressive mechanisms may compensate (Vermeersch et al., 2018). The GARP–αvβ8–TGF-β axis should therefore be interpreted as a spatially restricted and potentially actionable Treg mechanism, but not as a universal determinant of tumour tolerance.

CNS2/TSDR demethylation provides a molecular memory of the regulatory lineage. In committed Tregs, demethylated FOXP3 regulatory elements allow stable transcription-factor binding and help maintain FOXP3 expression through cell division. Induced or transient FOXP3^+^ cells that lack this epigenetic imprint are more prone to losing FOXP3 expression and suppressive function. FOXP3 expression should therefore not be equated with stable Treg identity unless it is supported by the appropriate chromatin state. This distinction is reinforced by evidence that Treg development requires two independent but complementary events: FOXP3 expression and establishment of a Treg-specific CpG hypomethylation pattern. Treg-type hypomethylation can be initiated by TCR stimulation independently of FOXP3, but is required for Foxp3^+^ cells to acquire Treg-type gene expression, lineage stability, full suppressive activity, and sustained expression of functional molecules such as CTLA-4 and CD25 (Ohkura et al., 2012). In tumour Tregs, repeated exposure to antigenic, cytokine, and metabolic signals makes this epigenetic infrastructure essential.

Several transcription factors connect environmental sensing to this stable programme. IL-2–STAT5 signalling supports FOXP3 expression, Treg survival, and lineage maintenance. NFAT links TCR-derived signals to FOXP3 enhancer activity, while RUNX–CBFβ contributes to FOXP3 stability through regulatory elements such as CNS2. This TCR–TGF-β integration is supported by enhancer-mapping studies showing that NFAT and Smad3 bind a conserved Foxp3 enhancer, cooperate to induce histone H4 acetylation, and are required for Foxp3 induction in response to TCR/CD3–CD28 and TGF-β signalling; Smad3 appears most important during early induction, whereas NFAT contributes to sustained enhancer activity and Foxp3 expression (Tone et al., 2008). BATF and IRF4 help sustain activated and tissue-adapted Treg programmes, whereas IKZF family members, including Helios, are embedded within the broader architecture of Treg identity and stability. This accessory transcription-factor network is essential because FOXP3 is necessary but not sufficient to specify the complete Treg programme; instead, Treg identity emerges from FOXP3 operating together with context-dependent transcriptional modules that control suppressive function, tissue adaptation, and lineage stability (Trujillo-Ochoa et al., 2023). TOX may participate in chronic-stimulation-associated T cell states in tumours, but its precise role in tumour-Treg stability remains less established.

Metabolism and epigenetics are deeply connected. Metabolites act as substrates or cofactors for chromatin-modifying enzymes: acetyl-CoA supports histone acetylation, S-adenosylmethionine supports histone and DNA methylation, α-ketoglutarate influences TET- and Jumonji-dependent demethylation, and NAD^+^ regulates sirtuin-dependent chromatin and protein deacetylation (Wellen et al., 2009; Mentch et al., 2015; Carey et al., 2015; Imai et al., 2000). Nutrient availability, lactate stress, mitochondrial activity, amino-acid catabolism, and lipid metabolism can therefore shape the epigenetic conditions under which FOXP3 and its partner transcription factors operate, including through TET-dependent stabilization of Foxp3 regulatory elements and acetylation-dependent control of FOXP3 protein stability (Yue et al., 2016; van Loosdregt et al., 2011; Beier et al., 2011). Conversely, FOXP3 and the Treg chromatin programme determine how these cells interpret metabolic stress. Mitochondrial stress may further connect metabolic fitness with durable inflammatory and epigenetic states. Mitochondrial dysfunction can alter respiration, redox balance, NAD^+^ availability and metabolite supply, while mitochondrial danger signals such as cytosolic mtDNA can activate cGAS–STING and NF-κB pathways, linking organelle damage to chronic inflammatory tone. Although this framework has been developed mainly in aging and inflammaging biology, it provides a useful conceptual bridge for tumour Treg niches, in which mitochondrial adaptation, redox stress and NAD^+^-dependent chromatin regulation may influence regulatory-cell persistence and suppressive stability (Bautista and López-Cortés, 2026a). The convergence of extracellular signalling, metabolic adaptation and epigenetic fixation in stabilizing the tumour-Treg programme is summarized in Figure 2B.

## Spatial Treg architecture in immunotherapy resistance: evidence and boundaries

Immunotherapy resistance is not a unitary Treg-mediated process. It can arise from tumour-cell-intrinsic defects in antigen presentation or interferon signalling, oncogenic immune-exclusion programmes, fibroblast and extracellular-matrix barriers, vascular dysfunction, suppressive myeloid states and regulatory lymphocytes. Spatial Treg niches should therefore be interpreted as context-dependent resistance modules embedded within a broader tissue architecture, rather than as universal, apical or non-redundant determinants of therapeutic failure.

For immune-checkpoint blockade, cancer vaccines, radiotherapy and adoptive cell therapy to succeed, tumour-reactive lymphocytes must be primed, enter malignant tissue, receive local antigenic restimulation, overcome suppressive signals and execute tumour-cell killing. Tregs can interfere with this sequence by limiting dendritic-cell costimulation, competing for cytokines and restraining effector-cell function (Tumeh et al., 2014; Mariathasan et al., 2018; Kamada et al., 2019). However, the coexistence of Tregs and ineffective or excluded effector cells does not establish that resistance is functionally Treg-dependent. A spatially defined regulatory compartment should initially be considered Treg-associated; stronger designations such as Treg-reinforced or Treg-dependent require evidence that Treg perturbation alters the local immune architecture and therapeutic response.

Direct human evidence linking spatial Treg architecture to immune-checkpoint-blockade outcome remains limited but is beginning to emerge. In a retrospective multiplexed CODEX analysis of pretreatment NSCLC specimens from patients subsequently treated with PD-1-axis immunotherapy, non-responding tumours showed greater Treg representation within stromal and peripheral tumour-margin compartments. Spatial interactions between Tregs and both CD8^+^ T cells and monocytes were also more frequent in non-responders, while increased Treg–monocyte interactions were associated with poorer survival (Monkman et al., 2024). These findings directly associate Treg localization and cellular neighbourhood with treatment outcome, but they derive from a small retrospective cohort and do not establish that Tregs were the dominant causal mechanism. They should therefore be regarded as treatment-linked human association requiring prospective and external validation, rather than as a validated predictive biomarker.

Primary resistance to PD-1 or PD-L1 blockade illustrates the importance of the broader effector architecture. PD-1 blockade is most effective when tumour-reactive CD8^+^ T cells are already present at the invasive margin or within tumour parenchyma and are restrained predominantly by the PD-1–PD-L1 axis. Responders to pembrolizumab in melanoma showed higher pretreatment densities of CD8^+^, PD-1^+^ and PD-L1^+^ cells at the invasive margin and within tumour parenchyma, closer PD-1–PD-L1 proximity and treatment-induced proliferation of intratumoral CD8^+^ T cells that correlated with tumour regression (Tumeh et al., 2014). This study establishes spatial effector-cell organization as a clinically relevant correlate of checkpoint response, but the response-associated population was the CD8^+^ compartment rather than Tregs. It therefore supports the broader topology-of-resistance framework, not a direct causal relationship between spatial Treg position and therapeutic outcome.

The predictive value of CD8^+^ infiltration also depends on functional state rather than abundance alone. In checkpoint-treated melanoma, responding lesions were enriched for a memory-like CD8^+^ T-cell state marked by TCF7, IL7R and reduced coinhibitory-receptor expression, whereas non-responding lesions were enriched for exhausted-like states expressing CD39/ENTPD1, TIM3/HAVCR2, PDCD1, LAG3 and CD38. TCF7^+^CD8^+^ T-cell frequency predicted response and improved survival in an independent anti-PD-1-treated cohort (Sade-Feldman et al., 2018). Activated and effector-memory T-cell populations have similarly been associated with response to anti-PD-1 alone or combined anti-PD-1 and anti-CTLA-4 therapy (Gide et al., 2019). When CD8^+^ T cells are absent, excluded, poorly primed or terminally dysfunctional, disruption of Tregs may be insufficient because the tumour lacks the recoverable effector substrate required for rejection.

Mechanistically, this recoverable compartment is now understood to include TCF1^+^PD-1^+^ stem-like or progenitor-exhausted CD8^+^ T cells that retain self-renewal capacity and generate more differentiated exhausted progeny. These cells, rather than terminally exhausted CD8^+^ T cells, constitute a principal cellular substrate for response to PD-1-pathway blockade and improved tumour control (Siddiqui et al., 2019; Miller et al., 2019). Treg-directed intervention is therefore most plausible when an addressable regulatory population coexists with antigen presentation and an anatomically accessible progenitor-exhausted CD8^+^ compartment capable of sustaining effector-cell replenishment.

Effector competence must also encompass tissue-resident memory CD8^+^ T cells (TRM), whose cellular state and retention within tumour-relevant compartments can provide information beyond total CD8^+^ T-cell abundance. Tumour TRM or TRM-like cells are commonly identified using combinations of CD69, CD103/ITGAE, CD49a/ITGA1 and CXCR6 together with cytotoxic, checkpoint and tissue-retention programmes; CD103 alone should not be treated as a universal or exclusive marker of residency. CD8^+^CD103^+^ tumour-infiltrating lymphocytes with resident-memory characteristics have been associated with prolonged survival in NSCLC, high-grade serous ovarian cancer and breast cancer (Djenidi et al., 2015; Webb et al., 2014; Savas et al., 2018). In analyses integrating single-cell data with tumour transcriptomes from atezolizumab trials in lung and bladder cancer, intratumoral CD103^+^CD8^+^ TRM were clonally expanded, expressed cytotoxic and checkpoint programmes and were associated with treatment outcome (Banchereau et al., 2021). Experimental cancer-vaccine studies further showed that induction of local TRM was required for optimal tumour control (Nizard et al., 2017). In selected tumour contexts, including gastric cancer, CXCL13^+^CD103^+^CD8^+^ TRM also participate in tertiary-lymphoid-structure-associated immune circuits linked to response to PD-1 blockade (Hu et al., 2024). These data provide a more directly established relationship between spatial effector residency and therapeutic outcome than is currently available for Treg localization.

TRM biology also demonstrates that TGF-β cannot be interpreted exclusively as a Treg-supportive or immunosuppressive signal. TGF-β contributes to CD103 induction and tissue retention in CD8^+^ T cells, whereas RUNX3 coordinates a broader transcriptional programme of residency in non-lymphoid tissues and tumours (Milner et al., 2017). Migratory dendritic cells expressing αvβ8 integrin can activate latent TGF-β and precondition CD8^+^ T cells for a TRM fate (Mani et al., 2019). Integrin-β8-expressing type 1 Tregs can similarly increase local TGF-β bioavailability and promote CD8^+^ TRM generation in infection models, although the general relevance of this mechanism to tumour TRM remains to be established (Ferreira et al., 2020). More recent tumour evidence indicates that Treg-derived TGF-β can also promote the differentiation and maintenance of tumour-infiltrating bystander CD8^+^ TRM-like cells through KLF2 repression (Lin et al., 2026). Although that study did not establish a specific requirement for GARP–αvβ8-mediated activation, it reinforces the principle that Treg-controlled TGF-β availability may either restrain antitumour effector function or support tissue residency according to the antigen specificity, differentiation state and anatomical position of the responding CD8^+^ population. Regulatory and resident-memory compartments should therefore not be assumed to be intrinsically antagonistic.

The effect of TGF-β depends on its cellular source, anatomical compartment, timing and the differentiation state of the responding CD8^+^ T cell. Local TGF-β can support CD103^+^ TRM generation and vaccine efficacy, as TGF-β blockade reduced TRM induction and tumour control following mucosal cancer vaccination (Nizard et al., 2017). Conversely, TGF-β-dependent lymphoid residency can retain stem-like tumour-specific CD8^+^ T cells in tumour-draining lymph nodes and limit their differentiation into migratory effectors; targeted interruption of this pathway facilitated effector-cell entry into tumours after vaccination (Li G. et al., 2022). In immune-excluded tumour models, combined PD-L1 and TGF-β blockade expanded stem-like CD8^+^ T cells, promoted their differentiation into non-exhausted IFN-γ-high effectors and increased effector-cell motility and accumulation within tumours (Castiglioni et al., 2023). TGF-β blockade should therefore not be expected to uniformly improve or impair TRM immunity: its net effect depends on whether tumour control requires preservation of functional local residency, release of lymphoid stem-like precursors, disruption of stromal exclusion or attenuation of Treg-supported suppression.

Complementary functional evidence for a spatial Treg resistance module has been obtained in KRAS-G12 C-mutant lung cancer. Spatial multiplex imaging identified therapy-induced cellular communities containing CD4^+^ and CD8^+^ T cells, dendritic cells and Tregs after KRAS-G12 C inhibition. Although treatment increased CD8^+^ T-cell activation and generated CXCL9-expressing dendritic-cell states, suppressive Tregs remained in frequent contact with effector T cells. Similar communities were detected in treatment-naive human lung-adenocarcinoma specimens, whereas evidence that Treg depletion improved the response to combined KRAS-G12C inhibition and anti-PD-1 was obtained in experimental models (Cole et al., 2024). These findings support a causal contribution of Tregs within a therapy-induced spatial immune community, while clearly separating the descriptive human evidence from the intervention-linked preclinical evidence.

Treg-rich immune-excluded tumours represent a particularly important boundary condition. These tumours may contain lymphocytes, but effector cells remain in stromal or peritumoural compartments rather than penetrating malignant nests. This phenotype should not be attributed exclusively to Tregs because fibroblast-derived TGF-β programmes, collagen-rich stroma, matrix-remodelling CAF states and vascular abnormalities can independently restrict T-cell entry. In metastatic urothelial cancer, lack of response to atezolizumab was associated with a fibroblast TGF-β-response signature, particularly when CD8^+^ T cells were retained within fibroblast- and collagen-rich peritumoural stroma. In experimental immune-excluded tumours, combined TGF-β and PD-L1 blockade reduced stromal TGF-β signalling and enabled T-cell penetration into the tumour centre (Mariathasan et al., 2018). Tregs may reinforce this architecture by limiting dendritic-cell function or local restimulation, but their presence should not be assumed to constitute its initiating or dominant cause.

Broader spatial studies provide important non-Treg-centred counterexamples. Imaging mass cytometry of treatment-naive, early-stage NSCLC in the TRACERx study identified four histology-independent tumour-microenvironment archetypes. Immune-low tumours were characterized by fibroblast barriers separating CD8^+^ T cells from malignant cells, whereas neutrophil-rich architectures were associated with metastatic evolution and shorter disease-free survival (Enfield et al., 2024). Because the cohort was treatment naive, these findings establish spatial determinants of immune surveillance and clinical outcome rather than direct predictors of checkpoint-blockade response. Nevertheless, they demonstrate that stromal and myeloid programmes can dominate immune failure without requiring a Treg-centred mechanism.

Longitudinal spatial multi-omics of melanoma treated with immune-checkpoint blockade similarly linked resistance to peri-tumoural lymphoid aggregates, limited intratumoral T-cell infiltration and emergent MITF^+^SPARCL1^+^ and CENPF^+^ malignant subclones (Quek et al., 2024). The study implicated malignant-cell evolution and broader tissue organization without identifying a dominant Treg-mediated mechanism. Together, these counterexamples show that spatial organization is relevant to therapeutic resistance, but the dominant topology may be malignant-cell, fibroblast, myeloid, vascular, effector-cell or Treg driven according to the tumour context.

Adaptive resistance provides stronger evidence that Tregs can become functionally relevant in selected settings. In advanced gastric cancer, PD-1^+^ effector Tregs were highly suppressive, expressed high levels of CTLA-4 and were enriched within tumours relative to peripheral blood. Patients who developed hyperprogressive disease after anti-PD-1 therapy showed increased tumour-infiltrating Ki-67^+^ effector Tregs after treatment, whereas these cells decreased in patients without hyperprogression. PD-1 blockade enhanced human Treg suppressive activity *in vitro*, and Treg-specific PD-1 deficiency or blockade in mice increased Treg proliferation, immunosuppression and tumour growth (Kamada et al., 2019). Anti-PD-1 treatment can also expand intratumoral follicular regulatory T cells concentrated within tertiary lymphoid structures, where they restrict antitumour responses in experimental models (Eschweiler et al., 2021). These observations support treatment-induced regulatory amplification but do not establish a universal pretreatment spatial Treg biomarker.

Resistance to CTLA-4 blockade also depends on the intratumoral regulatory context. In experimental systems, therapeutic efficacy is associated with an increased effector-T-cell-to-Treg ratio, including Fc-dependent reduction of CTLA-4-high tumour Tregs (Selby et al., 2013; Simpson et al., 2013). However, anti-CTLA-4 activity in humans cannot be reduced to Treg depletion, and clinical responses are not currently selected using spatial Treg biomarkers. Target expression, Fcγ-receptor engagement, effector-cell availability and tissue accessibility collectively determine whether checkpoint targeting produces local Treg depletion.

Treg-mediated impairment of dendritic-cell function constitutes another plausible resistance mechanism. Tregs can remove CD80 and CD86 from antigen-presenting cells through CTLA-4-dependent trans-endocytosis, weakening local costimulation and tumour-reactive T-cell priming or restimulation (Wing et al., 2008; Qureshi et al., 2011). Vaccination and radiotherapy may therefore expose tumour antigens without producing durable immunity when antigen-presenting interactions remain dominated by regulatory cells. Treg depletion has enhanced tumour-antigen-specific responses induced by dendritic-cell vaccination (Dannull et al., 2005), and stereotactic radiotherapy can increase functionally suppressive tumour-infiltrating Tregs (Muroyama et al., 2017). Nevertheless, these findings demonstrate regulatory constraint rather than validating a spatially defined Treg niche as a treatment-selection biomarker.

The available evidence therefore supports a graded hierarchy. First, spatial organization of the tumour microenvironment is firmly associated with immune surveillance and immunotherapy outcome. Second, human treatment-linked associations involving Treg localization and Treg-centred interactions are emerging but remain observational. Third, causal spatial Treg dependency is supported most directly by experimental perturbation studies. Fourth, multiple clinically relevant resistance architectures are driven predominantly by malignant, stromal, vascular or myeloid programmes rather than by Tregs. Fifth, CD8^+^ TRM provide comparatively stronger prognostic and treatment-linked evidence that the anatomical positioning of a defined T-cell state can influence clinical outcome, although their identification and predictive thresholds remain tumour-type and assay dependent.

To our knowledge, no prospective human study has yet simultaneously demonstrated a defined spatial Treg state, selective functional disruption of that state and improved therapeutic outcome. Spatial Treg localization should therefore not be presented as a universal determinant of resistance. Its translational value lies in identifying a candidate regulatory module whose relevance must be assessed together with tumour-intrinsic resistance programmes, stromal and vascular barriers, myeloid organization, antigen-presentation capacity and the presence of recoverable tumour-reactive effector cells, including intratumoral TRM and stem-like populations capable of sustaining or replenishing the effector response. A therapeutically actionable Treg niche requires convergent evidence of an activated tumour-Treg state, anatomically plausible interactions, functional Treg dependency, residual effector competence and treatment-linked niche remodelling.

## Therapeutic reprogramming and selective depletion of tumour Tregs

Therapeutically targeting tumour Tregs requires a shift from systemic immune deregulation to tumour-selective disruption of suppressive regulatory states. Because FOXP3^+^ Tregs are essential for peripheral tolerance, the goal is preferential interference with Tregs that have been adapted, expanded or stabilized within malignant tissue (Tanaka and Sakaguchi, 2019). In this framework, tumour-infiltrating Tregs may be selectively depleted, functionally reprogrammed or disabled while preserving tumour-reactive effector T cells and peripheral Treg compartments that prevent autoimmunity. Two therapeutic principles follow: selective depletion of tumour-enriched Treg populations and functional reprogramming of the signals that maintain their suppressive state. However, the strategies discussed below span markedly different levels of clinical maturity, ranging from established checkpoint inhibitors whose activity is not spatially guided or Treg selective, through early-phase Treg-depleting agents, to predominantly preclinical niche-reprogramming approaches. These distinctions are summarized in Table 2 together with niche-level mechanisms, optimal biological contexts and translational limitations. Representative ongoing and recently reported clinical programmes targeting CCR8, CD25, CTLA-4 and engineered IL-2 pathways are summarized in Table 3.

**TABLE 2:** Therapeutic strategies to disrupt tumour-associated Treg niches and their current evidence levels.

Strategy	Main target	Niche-level mechanism	Candidate biological context	Evidence	Translational limitation
CCR8-targeted antibodies	CCR8^+^ tumour Tregs	Fc-dependent depletion of tumour-enriched suppressive Tregs	Tumours enriched in activated CCR8^+^FOXP3^+^ Tregs with FcγR-competent effector cells	Early clinical development. Strong preclinical evidence; several agents are undergoing phase I or phase I/II evaluation, but none is approved or selected using a validated spatial biomarker (NCT05007782; NCT05537740; NCT05518045)	Clinical efficacy, tumour selectivity and immune toxicity remain to be established; requires effective intratumoral ADCC or ADCP
Fc-optimized anti-CD25	CD25	Preferential depletion of CD25-high Tregs while preserving IL-2 signalling in effector T cells	Treg-rich tumours with high CD25 expression and preserved effector-cell competence	Early clinical evidence. Preclinical activity was followed by phase I pharmacodynamic evidence of peripheral and intratumoral Treg depletion with RG6292, but clinical efficacy was insufficient in the evaluated populations	CD25 is also expressed by activated effector T cells; Treg depletion has not yet translated into consistent clinical benefit
Conventional anti-CTLA-4	CTLA-4 on activated T cells and Tregs	Checkpoint blockade, with a possible context-dependent contribution from Fc-mediated intratumoral Treg depletion	Tumours with pre-existing antitumour T-cell responses and CTLA-4-sensitive immune regulation	Approved therapy in selected cancers, but not approved as selective Treg depletion or spatially guided treatment	Immune-related toxicity; the contribution of Treg depletion to human clinical efficacy remains context dependent and is not prospectively measured
Fc-enhanced anti-CTLA-4	CTLA-4-high intratumoral Tregs and effector checkpoints	Enhanced FcγR engagement combined with checkpoint blockade and potential intratumoral Treg depletion	Immunologically resistant tumours retaining recruitable or reinvigoratable effector cells	Early clinical evidence. Botensilimab plus balstilimab has shown preliminary phase I activity, but is not approved and was not evaluated using spatial Treg-based patient selection	Non-randomized early-phase evidence; mechanism and predictive biomarkers require prospective validation
CCR4-targeted antibodies	CCR4-expressing malignant T cells and CCR4^+^ Tregs	Depletion of CCR4-expressing populations, including suppressive Tregs in some settings	CCR4-expressing cutaneous T-cell lymphoma; investigational use in Treg-rich solid tumours	Approved for selected cutaneous T-cell lymphomas, but not as Treg-directed therapy in solid tumours. Solid-tumour combinations have provided early pharmacodynamic evidence with limited or variable efficacy	Limited Treg specificity; depletion of CCR4^+^ Tregs has not consistently predicted tumour response
TGF-β blockade	TGF-β-driven stromal and regulatory programmes	Reduces stromal exclusion, fibroblast activation and regulatory immune tone	Immune-excluded, CAF-rich or TGF-β-high tumours	Investigational clinical strategy. Mechanistic and preclinical evidence is strong, but no therapy is approved specifically to disrupt a spatial Treg niche	Broad pathway activity, context-dependent toxicity and incomplete Treg specificity; spatial patient-selection criteria remain unvalidated
CD39/CD73/A2A targeting	Extracellular adenosine pathway	Reduces adenosine-mediated suppression and may restore effector-cell function	Adenosine-rich, hypoxic or metabolically suppressive tumours	Clinical investigation across several early-phase agents; spatial Treg-niche selection remains unvalidated	Pathway redundancy, heterogeneous target expression and uncertain predictive biomarkers
Ido1/kynurenine/AhR targeting	Tryptophan catabolism and AhR signalling	Disrupts kynurenine-supported regulatory and myeloid suppressive circuits	Ido1- or TDO-high tumours with evidence of kynurenine–AhR activity	Investigational. Ido1 inhibition has encountered major late-stage clinical failures; direct AhR-based Treg-niche reprogramming remains predominantly preclinical or early translational	Previous clinical failures, pathway redundancy and lack of validated patient-selection markers
Lactate and hypoxia modulation	Lactate transport and metabolism, HIF-dependent programmes	Reduces the metabolic fitness advantage of Tregs and may improve effector-cell access and function	Highly glycolytic, lactate-rich or hypoxic tumours	Predominantly preclinical or exploratory for Treg-niche disruption	Indirect Treg targeting, systemic metabolic effects and difficult spatial biomarker definition
IL-2 pathway tuning	IL-2/CD25/STAT5	Redirects cytokine support towards effector or memory cells while limiting Treg expansion	Tumours with preserved effector cells but IL-2-dependent regulatory dominance	Clinical development is ongoing for several engineered IL-2 and CD25-directed agents, but spatially guided Treg-niche application remains investigational	Agent-dependent receptor bias; poorly calibrated signalling can expand Tregs, impair effector cells or cause systemic toxicity
Spatial Treg-niche-guided treatment selection	Composite Treg state, spatial topology and functional immune competence	Matches selective depletion or niche reprogramming to the dominant causal architecture of immune suppression	Tumours with convergent evidence of activated Tregs, relevant positioning, functional dependence and residual effector competence	Future clinical hypothesis requiring prospective validation. No approved therapy is currently selected using a spatial Treg-niche biomarker	No standardized assay, threshold or validated treatment algorithm; requires multicentre analytical and clinical validation

**TABLE 3:** Representative ongoing and recently reported clinical programmes targeting CCR8, CD25, CTLA-4 and engineered IL-2 pathways.

Target	Agent and therapeutic design	Clinical setting	Phase and status	Trial
CCR8	Denikitug/GS-1811, afucosylated anti-CCR8 antibody, alone or with zimberelimab	Advanced solid tumours	Phase I; recruiting	NCT05007782
CCR8	CHS-114, afucosylated anti-CCR8 antibody, alone or with toripalimab	Advanced solid tumours, including HNSCC	Phase I; recruiting	NCT05635643
CCR8	BAY 3375968, Fc-optimized anti-CCR8 antibody, alone or with pembrolizumab	Selected advanced solid tumours	Phase I; active, not recruiting	NCT05537740
CD25	Vopikitug/RG6292, IL-2-sparing anti-CD25 Treg-depleting antibody, alone or with atezolizumab	Advanced solid tumours	Two phase I studies reported; development not continued in the evaluated populations	NCT04158583; NCT04642365
CD25	E7777, IL-2–diphtheria-toxin fusion targeting CD25-expressing cells, with pembrolizumab	Recurrent or metastatic solid tumours, including ovarian and MSI-H cancers	Phase I/II; active, not recruiting	NCT05200559
CTLA-4	Botensilimab, Fc-enhanced anti-CTLA-4 antibody, alone or with balstilimab	Refractory metastatic colorectal cancer	Phase II; active, not recruiting	NCT05608044
CTLA-4	Volrustomig, PD-1–CTLA-4 bispecific antibody	High-risk locally advanced cervical cancer after chemoradiotherapy	Phase III; recruiting	NCT06079671
IL-2 variant	STK-012, engineered partial IL-2 agonist designed to favour antigen-activated T cells	Advanced solid tumours and first-line NSCLC	Phase I/II; recruiting	NCT05098132
IL-2 variant	TransCon IL-2 β/γ, long-acting IL-2rβ/γ-selective prodrug	Locally advanced or metastatic solid tumours	Phase I/II; active, not recruiting	NCT05081609
IL-2 variant	GI-101/GI-101A, CD80–IgG4 Fc–IL-2-variant fusion, alone or in combination	Advanced or metastatic solid tumours	Phase I/II; recruiting	NCT04977453

### Selective depletion

CCR8 currently represents the strongest preclinical proof of concept for tumour-selective Treg targeting. Across multiple human and mouse tumours, CCR8 is preferentially enriched on activated tumour-infiltrating Tregs compared with circulating Tregs, conventional T cells and most non-malignant immune compartments (De Simone et al., 2016; Plitas et al., 2016; Whiteside et al., 2021; Kidani et al., 2022). Its therapeutic relevance therefore lies less in blocking CCR8 as a conventional chemokine-receptor pathway and more in exploiting CCR8 as a tumour-Treg-enriched surface address. In this framework, the key intervention is Fc-enabled elimination of CCR8^+^ tumour Tregs rather than simple interruption of CCR8–ligand signalling (Moreno Ayala et al., 2019).

This distinction is mechanistically important. Van Damme et al. showed that CCR8 marks a highly activated and strongly suppressive tumour-Treg population in both mouse and human tumours, but that antitumour efficacy depended on antibody format. An ADCC-deficient CCR8-blocking antibody produced limited antitumour activity, whereas an ADCC-competent CCR8-targeting antibody selectively depleted intratumoral Tregs, induced antitumour immunity and synergized with anti-PD-1 therapy (Van Damme et al., 2021). Consistent with this principle, Fc-optimized anti-CCR8 antibodies can deplete suppressive Tregs in human tumour models, while CCR8-targeted removal of clonally expanded tumour Tregs can unleash CD8^+^ effector responses and generate durable antitumour immunity (Campbell et al., 2021; Kidani et al., 2022).

Clinical translation of CCR8 targeting nevertheless remains at an early stage. Several anti-CCR8 antibodies, including denikitug, BAY 3375968, CHS-114, LM-108 and HC006, are being evaluated in phase I or phase I/II studies in advanced solid tumours (NCT05007782, NCT05537740, NCT05635643, NCT05518045 and NCT06304571). Preliminary denikitug data indicate pharmacodynamic depletion of intratumoral CCR8^+^ Tregs and early antitumour activity, but these findings remain non-randomized and require confirmation in larger studies (Bockorny et al., 2026). As of July 2026, the denikitug and CHS-114 studies remain recruiting, whereas the BAY 3375968 and LM-108 studies are active but no longer recruiting. CHS-114 is an afucosylated anti-CCR8 antibody designed to enhance Fc-dependent depletion of intratumoral Tregs, illustrating the central importance of antibody format in this therapeutic class (NCT05007782, NCT05537740, NCT05635643, NCT05518045) (Wang et al., 2026). CCR8 should therefore be described as a promising early clinical strategy rather than an established tumour-Treg therapy.

Early clinical findings are consistent with the need for both target-based and causal enrichment. In a pooled phase I/II analysis of cafelkibart/LM-108 combined with anti-PD-1 therapy in previously treated gastric cancer, the objective response rate was 36.1% among 36 efficacy-evaluable patients. Among 11 patients whose disease had progressed following first-line treatment, eight had high CCR8 expression; seven of these eight patients responded, corresponding to an exploratory objective response rate of 87.5% and a disease-control rate of 100% (Gong et al., 2024). These observations support further evaluation of CCR8-enriched populations but derive from a very small, non-randomized subgroup and do not establish that CCR8 expression alone identifies a Treg-dependent or immune-inflamed resistance architecture. Prospective studies must determine whether therapeutic benefit is independently predicted by CCR8-high tumour Tregs together with preserved antigen presentation, recoverable effector-cell competence and the absence of dominant tumour-intrinsic exclusion programmes.

CCR8 should therefore be regarded as a promising early clinical strategy and a marker of therapeutic addressability, rather than as an established therapy or a stand-alone biomarker of functional Treg dependency.

The efficacy of depleting antibodies depends on Fcγ-receptor biology. Productive engagement of Fcγ-receptor-expressing effector cells, including NK cells and macrophages, is required to mediate antibody-dependent cellular cytotoxicity or phagocytosis (Nimmerjahn and Ravetch, 2008). Antibody isotype, Fc engineering, glycosylation and the local balance between activating and inhibitory Fcγ receptors determine whether a Treg-targeting antibody merely binds its target or eliminates the suppressive population. This principle explains why Fc-optimized anti-CCR8, anti-CD25 and anti-CTLA-4 strategies can differ profoundly from non-depleting or poorly depleting antibodies despite recognizing the same nominal target. These Fc-dependent mechanisms are strongly supported experimentally, but their quantitative contribution to clinical activity in individual human tumours remains incompletely defined.

CD25 remains important but less tumour selective. Tregs express high CD25, but CD25 can also be expressed by recently activated effector T cells, and conventional anti-CD25 antibodies may deplete peripheral Tregs more efficiently than intratumoral Tregs. Fc optimization overcame intratumoral FcγRIIb-mediated resistance in preclinical models, enabling efficient depletion of tumour-infiltrating Tregs, increasing CD8^+^ effector T-cell-to-Treg ratios and synergizing with PD-1 blockade (Arce Vargas et al., 2017). Clinical translation has now moved beyond purely preclinical evaluation. The IL-2-sparing anti-CD25 antibody vopikitug/RG6292 produced sustained dose-dependent peripheral Treg depletion and measurable intratumoral Treg reduction in two phase I studies, both as monotherapy and with atezolizumab. However, objective responses were uncommon and the observed pharmacodynamic activity was insufficient to support further development in the evaluated solid-tumour populations (NCT04158583, NCT04642365) (Gambardella et al., 2025). An ongoing phase I/II study is also evaluating E7777, an IL-2–diphtheria-toxin fusion that targets CD25-expressing cells, in combination with pembrolizumab in recurrent or metastatic solid tumours (NCT05200559) (Mahdi et al., 2023). CD25-directed depletion should therefore be considered clinically tested but not clinically validated as an effective tumour-selective Treg strategy.

CTLA-4-directed therapy occupies a different level of evidence. Ipilimumab and tremelimumab are approved immune-checkpoint inhibitors in selected malignancies, establishing the clinical value of CTLA-4-directed therapy (Hodi et al., 2010; Patel et al., 2024). Their approvals, however, are not based on selective Treg depletion or the presence of a spatial Treg niche. Preclinical studies indicate that anti-CTLA-4 activity can involve Fcγ-receptor-mediated depletion of CTLA-4-high intratumoral Tregs, increasing the local effector-T-cell-to-Treg ratio in addition to relieving checkpoint inhibition (Selby et al., 2013; Simpson et al., 2013). The extent to which this mechanism explains clinical responses in humans is context dependent and remains less firmly established than the therapeutic efficacy of checkpoint blockade itself. Approved anti-CTLA-4 therapy should therefore not be equated with validated spatially guided Treg depletion.

The Fc-enhanced anti-CTLA-4 antibody botensilimab provides emerging clinical evidence for a strategy designed to extend activity in immunologically resistant tumours. Botensilimab combined with the anti-PD-1 antibody balstilimab demonstrated preliminary activity in a phase I study of heavily pretreated microsatellite-stable metastatic colorectal cancer (Bullock et al., 2024). These findings represent early clinical evidence rather than definitive validation: the study was non-randomized, treatment was not selected through a spatial Treg biomarker, and confirmatory phase II evaluation remains ongoing (NCT05608044). The randomized phase II study is active but no longer recruiting as of July 2026. A complementary strategy is represented by volrustomig, a PD-1–CTLA-4 bispecific antibody designed to concentrate CTLA-4 targeting within PD-1-expressing T-cell populations. Volrustomig has progressed to a recruiting phase III study in high-risk locally advanced cervical cancer, although its mechanism should be interpreted as dual-checkpoint modulation rather than selective Treg depletion (NCT06079671) (Tran et al., 2026).

CCR4 provides another clinically relevant but less selective example. Anti-CCR4 can reduce suppressive FOXP3^high^ Tregs and augment tumour-antigen-specific immune responses, but CCR4 is also expressed by other immune and malignant-cell populations (Sugiyama et al., 2013). Mogamulizumab is approved for relapsed or refractory mycosis fungoides and Sézary syndrome, where its therapeutic indication is directed against CCR4-expressing malignant T cells rather than spatially defined Treg niches (Kim et al., 2018; Kasamon et al., 2019). Early studies combining mogamulizumab with nivolumab, durvalumab or tremelimumab in solid tumours demonstrated peripheral and, in some cases, intratumoral Treg depletion, but clinical activity was variable or limited and did not consistently correlate with the extent of Treg reduction (Doi et al., 2019; Zamarin et al., 2020). These findings demonstrate that pharmacodynamic Treg depletion is achievable in patients but is not, by itself, sufficient to establish clinical benefit.

Combination checkpoint blockade can extend Treg-directed mechanisms into broader immune remodelling. In B16 melanoma models, combined CTLA-4 and PD-1 blockade with Flt3-ligand vaccination produced stronger tumour rejection than either pathway alone, increased effector T-cell infiltration, improved intratumoral effector-T-cell-to-Treg ratios and reduced the relative dominance of myeloid-derived suppressor cells (Curran et al., 2010). Such experiments provide mechanistic support for combination strategies, but they should be identified as preclinical unless supported by corresponding human trial evidence.

### Functional reprogramming

Depletion is only one route to therapeutic disruption. A complementary strategy is to interfere with the molecular and environmental programmes that stabilize suppressive Treg states or enable their local function. These interventions should be distinguished from direct Treg targeting because most act simultaneously on malignant, stromal and multiple immune compartments.

TGF-β exemplifies this distinction. Stromal and tumour-cell TGF-β signalling can promote fibroblast activation, extracellular-matrix remodelling and retention of effector lymphocytes outside malignant nests, thereby limiting the efficacy of PD-1 or PD-L1 blockade in immune-excluded tumours (Mariathasan et al., 2018; Tauriello et al., 2018). However, total TGF-β abundance does not identify where or by which cell the cytokine becomes biologically active. Activated Tregs can present latent TGF-β1 through GARP/LRRC32 and activate it through integrin αvβ8, generating a spatially restricted suppressive signal near responding lymphocytes (Tran et al., 2009; Worthington et al., 2015; Liénart et al., 2018). In experimental melanoma and breast cancer, Treg-specific Itgb8 deletion reduced intratumoral TGF-β signalling, restored CD8^+^ T-cell function and improved tumour control (Lainé et al., 2021). This identifies latent-TGF-β activation, rather than cytokine abundance alone, as a potentially actionable Treg dependency.

Therapeutic interpretation nevertheless requires caution. TGF-β can also support CD103 induction and CD8^+^ tissue-resident memory differentiation, and integrin-β8-expressing Tregs can promote TRM formation in infection models (Ferreira et al., 2020). Moreover, GARP loss does not impair tumour progression in every experimental system, indicating potential compensation by alternative activating cells or suppressive pathways (Vermeersch et al., 2018). Broad TGF-β inhibition, GARP–latent-TGF-β blockade and αvβ8 inhibition should therefore be regarded as biologically distinct strategies. Patient selection may require spatial assessment of GARP, ITGB8, active TGF-β, phosphorylated SMAD2/3, stromal exclusion and neighbouring CD8^+^ differentiation states rather than reliance on a generic TGF-β signature.

Adenosine-targeted therapy provides a second form of reprogramming. The CD39–CD73 axis converts extracellular ATP into adenosine, replacing an inflammatory danger signal with an immunosuppressive metabolite that restrains antitumour T-cell activity (Deaglio et al., 2007; Ohta et al., 2006). Blocking CD39, CD73 or adenosine A2A-receptor signalling may reduce this metabolic layer of suppression and improve effector-cell function (Beavis et al., 2017; Giuffrida et al., 2021). Similarly, IDO1–kynurenine–AhR targeting aims to prevent tryptophan catabolism from reinforcing regulatory immunity and suppressive macrophage states; in experimental IDO- or TDO-expressing tumours, AhR blockade can disrupt a kynurenine-induced Treg–macrophage axis and improve responses to PD-1 blockade (Campesato et al., 2020). These pathways are therapeutically investigational, but current evidence does not establish that spatially defined Treg dependence predicts clinical response to their inhibition.

Lactate and hypoxia modulation should be framed as niche reprogramming rather than direct Treg targeting. Tumour Tregs can remain functional in low-glucose, lactate-rich environments that impair effector lymphocytes because FOXP3 rewires T-cell metabolism towards oxidative phosphorylation and resistance to lactate-associated stress (Angelin et al., 2017). Tumour-infiltrating Tregs can also use lactic acid through MCT1-dependent uptake, supporting suppressive function within the tumour microenvironment (Watson et al., 2021). In highly glycolytic tumours, lactic acid can increase PD-1 expression on Tregs, potentially shifting the balance of PD-1 blockade towards regulatory rather than effector-cell activation (Kumagai et al., 2022). Hypoxia adds a spatial dimension by inducing Treg-recruiting chemokines such as CCL28 (Facciabene et al., 2011). Strategies targeting lactate transport, hypoxia or associated metabolic adaptations remain predominantly preclinical or exploratory with respect to selective Treg-niche disruption.

The IL-2 axis also requires therapeutic tuning. Tregs constitutively express high CD25 and depend on IL-2–STAT5 signalling for peripheral maintenance, FOXP3 stability and suppressive fitness, while limiting IL-2 availability for competing effector cells (Chinen et al., 2016; Setoguchi et al., 2005). Reprogramming this axis means redirecting cytokine support away from suppressive Treg maintenance and towards productive effector or memory T-cell responses. Next-generation CD25-targeting antibodies have been designed to deplete Tregs while preserving IL-2 signalling on effector T cells (Solomon et al., 2020). However, the ability of these agents to selectively remodel human tumour Treg niches without compromising activated effector cells or systemic tolerance remains under clinical investigation.

Current clinical programmes illustrate several distinct IL-2-engineering strategies. STK-012 is a partial IL-2 agonist designed to preferentially signal through CD25/CD122 on antigen-activated T cells while limiting broad peripheral immune activation and is being evaluated in a recruiting phase I/II study, including a randomized first-line NSCLC component (NCT05098132). TransCon IL-2 β/γ is a long-acting prodrug that releases an IL-2Rβ/γ-selective variant with minimal IL-2Rα engagement and is undergoing phase I/II evaluation as monotherapy and in combination regimens (NCT05081609) (Rosen et al., 2022). GI-101/GI-101A combines a CD80 ectodomain with an IgG4 Fc-linked IL-2 variant and is being evaluated alone and with pembrolizumab or other treatments in a recruiting phase I/II study (NCT04977453). These agents seek to redistribute IL-2 activity towards antitumour lymphocytes, but none has yet demonstrated that a spatially defined Treg niche predicts clinical benefit.

Costimulatory and coinhibitory receptors offer additional ways to alter Treg function. OX40 and GITR can modulate Treg suppressive activity, stability or intratumoral abundance, but their effects depend strongly on antibody format, Fcγ-receptor engagement and tumour context (Piconese et al., 2008; Bulliard et al., 2013). ICOS targeting can remodel the intratumoral cytotoxic-to-regulatory T-cell balance, whereas TIGIT signalling may act through both regulatory and exhausted effector compartments (Sainson et al., 2020). Broader Treg-associated targets, including LAG-3, TIM-3, VISTA, IGSF8, 4-1BB, CD40 and CD27, should therefore be interpreted as context-dependent immunomodulatory levers rather than Treg-specific switches (Bautista et al., 2026b). For most of these targets, any contribution of spatial Treg-niche disruption to clinical efficacy remains hypothetical and cannot be inferred from target expression alone.

Finally, metabolic and epigenetic destabilization represents a deeper therapeutic concept. Tumor Tregs persist because FOXP3-dependent transcription, chromatin accessibility, mitochondrial adaptation, lipid metabolism and immunosuppressive metabolites reinforce one another. Stable regulatory identity requires more than FOXP3 expression alone; it depends on epigenetic fixation of the Treg programme, including demethylation of conserved regulatory elements within the FOXP3 locus and broader Treg-type DNA hypomethylation patterns (Floess et al., 2007; Ohkura et al., 2012). Disrupting this integrated state could weaken suppressive function without complete cellular depletion. At present, however, selective metabolic or epigenetic destabilization of tumour Tregs remains a future therapeutic hypothesis because the same pathways frequently support other immune and non-immune populations and have not been clinically validated using spatial Treg biomarkers.

### Clinical maturity and translational boundary

No cancer therapy is currently approved on the basis of a spatial Treg-niche biomarker, and no prospectively validated spatial assay is used to select patients for Treg depletion or functional reprogramming. Spatial biomarkers therefore remain primarily discovery, pharmacodynamic or early translational tools. Approved CTLA-4 inhibitors validate checkpoint blockade, but not spatially guided or selectively Treg-depleting treatment (Hodi et al., 2010; Patel et al., 2024). Similarly, mogamulizumab validates CCR4 targeting in cutaneous T-cell lymphomas, not Treg-directed therapy in solid tumours (Kasamon et al., 2019).

The therapeutic landscape has nevertheless advanced beyond early preclinical development. Cafelkibart/LM-108 has entered a randomized phase III trial with toripalimab in CCR8-positive, second-line gastric or gastro-oesophageal-junction adenocarcinoma (NCT07362186). CHS-114 has also provided human pharmacodynamic proof of mechanism: paired HNSCC biopsies showed selective depletion of CCR8^+^ intratumoral Tregs, relative preservation of CCR8^-^ Tregs and a marked increase in the intratumoral CD8^+^-T-cell-to-Treg ratio (NCT05635643) (Wang et al., 2026). These findings establish that Fc-enabled CCR8 targeting can remodel the human tumour immune compartment, although clinical benefit and predictive biomarker validity remain unproven.

LAG-3 blockade provides a complementary example of context-dependent therapeutic activity. Nivolumab plus relatlimab improved progression-free survival in advanced melanoma in RELATIVITY-047, establishing LAG-3 as a clinically validated checkpoint (Tawbi et al., 2022). By contrast, RELATIVITY-098 showed no recurrence-free-survival benefit in completely resected melanoma compared with nivolumab alone (hazard ratio, 1.01) (Long et al., 2025). Although the adjuvant study did not directly test spatial target localization, the contrasting outcomes support the principle that efficacy depends on the availability of an appropriate tumour-associated, target-bearing immune substrate rather than target expression alone.

Other strategies remain at earlier levels of evidence. Botensilimab is undergoing phase II evaluation, CD25-directed treatment has demonstrated Treg depletion without sufficient clinical activity to validate depletion alone, and engineered IL-2, metabolic and stromal approaches remain investigational with respect to spatially defined Treg dependencies (Bullock et al., 2024; Gambardella et al., 2025).

The framework proposed here should therefore be regarded as a strategy for prospectively testing patient-selection hypotheses rather than as a validated clinical algorithm. Target expression, pharmacodynamic niche remodelling and clinical efficacy are distinct evidentiary levels. Future trials must establish whether spatial Treg states predict selective target engagement, restoration of effector immunity, treatment benefit and toxicity beyond conventional biomarkers.

## Current limitations of spatial technologies for Treg-niche analysis

Spatial technologies provide essential information about tissue organization, but they do not yet offer a direct or platform-independent measurement of Treg-mediated suppression. Sequencing-based spatial transcriptomic methods involve trade-offs among transcriptome coverage, molecular sensitivity, spatial resolution and tissue area. Spot-based platforms may capture transcripts from multiple adjacent cells, whereas higher-resolution sequencing methods can remain affected by incomplete transcript capture, molecular diffusion and gene-detection biases. Direct cross-platform comparisons have demonstrated substantial variation in capture efficiency, effective resolution, molecular diffusion and downstream biological interpretation across sequencing-based spatial transcriptomic technologies (You et al., 2024). Imaging-based spatial transcriptomic platforms can achieve cellular or subcellular resolution, but generally rely on predefined gene panels and remain sensitive to detection efficiency, signal contamination and cell-segmentation accuracy. Consequently, failure to detect a Treg marker or suppressive programme should not automatically be interpreted as biological absence.

Multiplex protein-imaging platforms provide single-cell or subcellular localization but are constrained by predefined marker panels and depend on antibody specificity, epitope preservation, staining efficiency, background correction, image registration and accurate cell segmentation. Segmentation or phenotyping errors can alter cell identities, intercellular distances and neighbourhood assignments, thereby affecting the inferred composition of a Treg niche. Reproducibility therefore requires validated reagents, standardized tissue processing, reference controls and harmonized analytical pipelines (Eng et al., 2022; Schapiro et al., 2022a). No single widely adopted platform simultaneously provides unbiased transcriptome-wide coverage, deep protein phenotyping, subcellular resolution, broad tissue coverage and straightforward compatibility with routine clinical workflows.

Computational analysis introduces an additional source of uncertainty. Cell-type deconvolution, transcript imputation, spatial clustering, neighbourhood detection and ligand–receptor inference are method dependent, and their performance varies according to tissue architecture, sequencing depth, reference datasets and analytical parameters (Li B. et al., 2022). High-dimensional spatial datasets also require substantial data storage, computational infrastructure and specialized expertise. Results obtained using different platforms or pipelines should therefore not be considered directly interchangeable without analytical benchmarking and orthogonal validation.

Most importantly for Treg-niche interpretation, spatial colocalization does not directly demonstrate functional suppression. Proximity between Tregs and CD8^+^ T cells, dendritic cells, macrophages or fibroblasts may reflect a causal interaction, but it may also result from shared recruitment signals, anatomical boundaries or upstream malignant and stromal programmes. Transcript abundance does not establish protein expression, receptor engagement, cytokine availability or suppressive activity. Candidate Treg niches should therefore be validated through complementary protein measurements, functional perturbation, *ex vivo* or organotypic models and, where possible, longitudinal biopsies obtained before and during treatment. Spatial association alone should be considered hypothesis-generating with respect to causality until local Treg dependency is demonstrated experimentally.

Clinical implementation remains limited by assay cost, specialized instrumentation, tissue requirements, restricted capture areas, turnaround time and the absence of standardized thresholds for defining clinically actionable spatial patterns. Current commercial platforms can also differ substantially in affordability, tissue coverage and technical performance, complicating multicentre validation (You et al., 2024; Schroeder et al., 2025). Before a spatial Treg biomarker can be used for patient selection, it will require compatibility with routinely available specimens, standardized pre-analytical procedures, locked analytical pipelines, inter-laboratory reproducibility and prospective validation against therapeutic response and toxicity. For Treg-niche biomarkers, current spatial platforms should therefore be regarded predominantly as discovery and pharmacodynamic tools until analytically validated and clinically deployable assays are established. Clinical translation should prioritize reduced, technically robust marker panels derived from spatially and functionally validated regulatory circuits.

## Biomarkers and a practical pipeline for spatial patient stratification

The clinical translation of Treg-directed therapy will depend on identifying patients whose tumours are Treg-niche dependent. This distinction is essential because FOXP3^+^ cell density can be prognostic in some settings, but is not by itself a predictive biomarker for Treg depletion or Treg reprogramming. The clinical meaning of FOXP3^+^ infiltration is context dependent: high FOXP3^+^ T-cell density has been associated with poor outcome in several malignancies, but with favourable prognosis in selected settings such as colorectal cancer, head and neck cancer, and some lymphomas, where regulatory cells may restrain tumour-promoting inflammation rather than suppress protective antitumour immunity. This ambiguity is further illustrated in colorectal cancer, where FOXP3 expression by cancer cells, rather than by tumour-infiltrating Tregs, correlated with disease progression and poor survival, underscoring the need for cell-type-resolved interpretation of FOXP3-based biomarkers (Kim et al., 2013; Frydrychowicz et al., 2017). Patient selection must therefore establish not only whether activated suppressive Tregs are present and spatially organized, but whether they constitute a causal and therapeutically reversible component of immune resistance (Table 4).

**TABLE 4:** Practical biomarker pipeline for spatially guided Treg immunotherapy.

Stage	Candidate biomarkers or measurements	Preferred platform	Purpose and validation
Tissue qualification and screening	Pan-cytokeratin, CD3, CD4, CD8, FOXP3, CD25, CCR8, CTLA-4 and Ki-67; optional TIGIT, ICOS and myeloid, stromal or vascular markers	Pathological review with multiplex immunofluorescence or serial immunohistochemistry on FFPE tissue	Confirms specimen quality and identifies activated, potentially targetable tumour Tregs using standardized processing, validated reagents and predefined phenotyping rules
Spatial confirmation	Compartment-specific Treg density, target-positive Treg frequency, CD8/Treg ratio, Treg–CD8 and Treg–DC proximity, tumour–stroma enrichment and FcγR^+^ effector-cell proximity	Multiplex imaging, MIBI, CODEX, imaging mass cytometry or targeted spatial transcriptomics	Determines whether Tregs occupy a plausible suppressive niche; requires prespecified regions, segmentation, neighbourhood definitions and distance thresholds
Mechanistic confirmation	CCR8, CTLA4, TIGIT, ICOS, IL2RA, LAYN, MAGEH1 and IL1R2; antigen-presentation, IFN-γ, TGF-β, myeloid and stromal programmes	Transcriptomics with orthogonal flow-cytometric, explant or functional assays	Distinguishes functional tumour-adapted Treg dependency from isolated FOXP3 expression or spatial colocalization
Provisional patient classification	Targetable Tregs, spatial suppressive circuitry, residual effector competence and therapy-compatible mechanisms, including FcγR^+^ macrophages or NK cells for Fc-dependent depletion	Integrated prespecified classifier	Classifies tumours as provisionally Treg-niche dependent, Treg-associated or non-actionable; thresholds must be tumour- and therapy-specific and externally validated
Clinical validation and deployment	Assay sensitivity, specificity, repeatability, inter-laboratory agreement, target engagement, niche remodelling, response and toxicity	Independent cohorts, prospective trials and a reduced FFPE-compatible multiplex assay	Establishes analytical reproducibility, clinical validity and utility while assessing tissue requirements, cost, turnaround time and feasibility for routine pathology

Causal architecture should constitute the first diagnostic layer. Before Treg phenotype, transcriptional state or spatial position is interpreted, the dominant mechanism of immune failure must be identified. Tumour-cell-intrinsic programmes such as WNT–β-catenin activation, loss of antigen presentation, defective IFN-γ–JAK signalling or failure to recruit cross-presenting dendritic cells can generate immune exclusion independently of Tregs (Spranger et al., 2015; Zaretsky et al., 2016; Shin et al., 2017; Jerby-Arnon et al., 2018). In tumours lacking antigen presentation, productive DC–T-cell priming or a recoverable effector compartment, the presence of FOXP3^+^ or CCR8^+^ cells does not establish that regulatory-cell depletion will restore antitumour immunity. Conversely, activated tumour Tregs embedded within an antigen-presenting, T-cell-inflamed and functionally recoverable microenvironment are more likely to constitute an actionable suppressive dependency.

This causal gate separates three biologically distinct configurations. In Treg-dependent tumours, regulatory cells execute a dominant suppressive mechanism, and their selective depletion or functional reprogramming is predicted to restore local immunity. In Treg-reinforced tumours, an upstream malignant, stromal, vascular or myeloid programme initiates resistance, but Tregs become functionally necessary amplifiers; these tumours are more likely to require combined targeting of both the initiating programme and the downstream regulatory compartment. In Treg-associated tumours, Tregs accompany an immune-excluded or dysfunctional architecture without maintaining its principal mechanism, making isolated Treg-directed therapy unlikely to succeed (Spranger et al., 2015; Glasner et al., 2023; Torres-Mejia et al., 2025). Upstream tumour-cell causality and downstream Treg dependency are therefore not mutually exclusive, but they must be distinguished experimentally rather than inferred from regulatory-cell abundance.

Pan-cancer immunogenomic analyses have identified recurrent immune states, including IFN-γ-dominant, inflammatory, lymphocyte-depleted and TGF-β-dominant tumours, with distinct leukocyte composition, macrophage balance and immunoregulatory programmes (Thorsson et al., 2018). These states provide biological context but do not independently establish causality. A tumour containing activated Tregs within an inflamed, antigen-presenting and CD8^+^-competent microenvironment is biologically different from a Treg-rich tumour dominated by tumour-cell-intrinsic exclusion, irreversible antigen-presentation failure, fibroblast-mediated restriction, suppressive myeloid states or lymphocyte depletion.

### Cellular biomarkers

Once the causal architecture has been assessed, the second diagnostic layer is Treg phenotype and therapeutic addressability. FOXP3 and CD25 define the canonical regulatory compartment but do not distinguish resting, bystander, transiently FOXP3-expressing or tumour-adapted Tregs (Sakaguchi et al., 1995; Saito et al., 2016). Human tumour-infiltrating Tregs frequently express activated regulatory programmes involving CTLA-4, TIGIT and ICOS together with tumour-enriched markers such as CCR8, LAYN, MAGEH1 and IL1R2 (De Simone et al., 2016; Plitas et al., 2016). CCR8 is particularly relevant because it identifies an activated tumour-enriched Treg population that can be exploited by Fc-enabled depleting antibodies (Van Damme et al., 2021). Ki-67 provides evidence of local proliferation, whereas Helios may support phenotypic characterization but should not be interpreted as a stand-alone marker of thymic origin or functional stability. A clinically useful cellular panel should therefore assess FOXP3, CD25, CTLA-4, CCR8, TIGIT, ICOS and Ki-67 together with CD4, CD8, malignant-cell, myeloid and stromal markers in the same specimen.

The clinical question at this level is targetability rather than causality. A tumour containing abundant CCR8^+^FOXP3^+^CD25^+^CTLA-4^+^ Tregs may provide a more accessible target for depletion than one with low CCR8 expression or diffuse FOXP3^+^ cells lacking activation markers (De Simone et al., 2016; Van Damme et al., 2021). However, target expression alone cannot establish that removal of the target-positive population will reverse the dominant resistance mechanism. High CTLA-4 or CD25 expression is similarly informative only when sufficiently enriched within the suppressive Treg compartment rather than broadly shared with recently activated effector cells. Cellular biomarkers therefore define therapeutic addressability, whereas causal and functional evidence defines therapeutic rationale.

Early clinical observations support target-based enrichment but do not yet validate CCR8 as a stand-alone causal biomarker. In pooled phase I/II analyses of cafelkibart/LM-108 combined with anti-PD-1 therapy in previously treated gastric cancer, encouraging antitumour activity was reported, including a high response rate in a very small exploratory subgroup with high CCR8 expression (Gong et al., 2024). These data are consistent with the possibility that CCR8-high tumours contain a therapeutically addressable regulatory compartment. However, the subgroup was non-randomized, comprised only eight patients and was not prospectively selected using an integrated classifier of immune inflammation, WNT–β-catenin activity, antigen-presentation competence or functional Treg dependency. The findings therefore support prospective evaluation of CCR8-enriched populations but do not demonstrate that CCR8 expression alone distinguishes Treg-dependent resistance from correlated regulatory infiltration.

### Transcriptomic biomarkers

The third diagnostic layer is the regulatory transcriptional state. Bulk RNA sequencing, single-cell RNA sequencing and spatial transcriptomics can determine whether phenotypically identified Tregs carry a coherent tumour-adapted suppressive programme. *CCR8*-centred signatures involving *LAYN, MAGEH1 and IL1R2,* interpreted together with *CTLA4, TIGIT, ICOS, IL2RA* and proliferation-associated transcripts, can help distinguish activated tumour Tregs from circulating-like, resting or transiently activated states.

Cellular resolution is essential because FOXP3 transcripts and protein are not confined to Tregs. Malignant cells from several tumour types can express FOXP3, and tumour-associated splice variants have also been described (Karanikas et al., 2008; Ebert et al., 2008). Bulk FOXP3 expression may therefore conflate malignant-cell transcription with regulatory-cell infiltration.

The clinical question at this level is whether a coherent suppressive programme exists, not whether FOXP3 or CCR8 is merely detectable. Strong tumour-Treg signatures embedded within an antigen-exposed and effector-competent microenvironment may support regulatory dependence. By contrast, weak Treg activation, absent suppressive modules or dominance of tumour-intrinsic, stromal or myeloid resistance programmes argues against isolated Treg-directed therapy.

Transcriptomic analysis should therefore evaluate both sides of the causal comparison: evidence supporting active Treg-mediated suppression and evidence identifying competing mechanisms that may dominate immune failure. Candidate exclusionary or combination-directing features include WNT–β-catenin-associated immune exclusion, B2M or MHC class I loss, defective IFN-γ–JAK signalling, deficient cDC1 recruitment and malignant-cell programmes associated with T-cell exclusion (Spranger et al., 2015; Zaretsky et al., 2016; Shin et al., 2017; Jerby-Arnon et al., 2018).

### Spatial biomarkers

The fourth diagnostic layer is tissue organization. Treg-directed therapy should be prioritized only when therapeutically addressable Tregs occupy anatomically plausible suppressive circuits. Treg–CD8 distance can distinguish potential short-range effector restraint from spatial segregation. Treg proximity to DCs may indicate regulation of antigen presentation or local restimulation, whereas proximity to macrophages, cancer-associated fibroblasts or collagen-rich stroma may identify myeloid or stromal regulatory circuits. Treg enrichment within inflamed tumour nests may indicate restraint of an ongoing immune response, whereas enrichment at invasive margins, perivascular regions or stromal corridors may reflect immune gating or secondary accumulation within an upstream exclusion programme (Barua et al., 2018).

A Treg-rich tumour is therefore not automatically Treg-niche positive. Multiplex imaging, MIBI, CODEX, imaging mass cytometry and spatial transcriptomics can identify candidate suppressive architectures, but proximity alone cannot determine whether Tregs initiate, reinforce or merely accompany them. Structured tumour–immune borders and multicellular communities have been associated with clinical outcomes, supporting spatial analysis as a source of biologically relevant information (Keren et al., 2018; Jackson et al., 2020). Nevertheless, spatial biomarkers must be interpreted alongside malignant-cell programmes and orthogonal evidence of functional dependency.

Spatial exclusion should not itself be interpreted as an indication for Treg depletion. In WNT–β-catenin-active tumours, for example, deficient recruitment of BATF3-lineage DCs and failure of spontaneous T-cell priming precede any potential Treg contribution (Spranger et al., 2015). A spatially organized Treg population within such a tumour may therefore be a secondary correlate rather than the mechanism maintaining immune exclusion. By contrast, Tregs positioned within an inflamed tumour containing recoverable CD8^+^ T cells and competent antigen-presenting cells provide a more plausible substrate for direct regulatory dependency (Tumeh et al., 2014; Sade-Feldman et al., 2018).

### Functional biomarkers

The fifth diagnostic layer is functional recoverability. The CD8/Treg ratio provides an interpretable measure of regulatory balance but does not establish whether the CD8^+^ compartment can be restored. In early lung adenocarcinoma, a reduced effector-CD8^+^-cell-to-Treg ratio reflects both loss of granzyme-B^+^ and IFN-γ^+^ CD8^+^ cells and expansion of activated CD39^hi^CD38^hi^PD-1^hi^CTLA-4^hi^FOXP3^hi^ Tregs (Lavin et al., 2017). The ratio should therefore be interpreted together with antigen-presentation competence, IFN-γ-associated inflammation, tumour-reactive or memory-like CD8^+^ states, terminal dysfunction, stromal exclusion and myeloid suppression.

Response to checkpoint blockade has been associated with TCF7^+^ memory-like CD8^+^ T-cell states, whereas non-response is enriched for more terminally dysfunctional states expressing CD39, TIM-3, PD-1, LAG-3 and CD38 (Sade-Feldman et al., 2018). A tumour containing activated Tregs, preserved antigen presentation and a reinvigoratable effector population is more suitable for Treg-directed intervention than a tumour lacking DC recruitment, antigen presentation or recoverable effector cells (Spranger et al., 2015; Sade-Feldman et al., 2018; Tumeh et al., 2014). Dominant macrophage, MDSC, fibroblast or vascular suppression may instead indicate a Treg-reinforced architecture requiring combination therapy.

For depleting antibodies, the Fcγ-receptor landscape is an intervention-specific biomarker. Target expression defines which cells can be bound, but depletion requires macrophages or NK cells capable of mediating antibody-dependent cellular cytotoxicity or phagocytosis. Activating and inhibitory Fcγ-receptor expression, effector-cell abundance and spatial proximity between FcγR-expressing cells and target-positive Tregs should therefore be incorporated into trials of Fc-engineered antibodies (Nimmerjahn and Ravetch, 2008; Simpson et al., 2013; Arce Vargas et al., 2017). Target expression defines accessibility; FcγR-enabled effector capacity determines whether depletion is biologically feasible.

An integrated patient-stratification framework should consequently ask five sequential diagnostic questions. First, what is the dominant causal architecture of immune resistance: Treg dependent, Treg reinforced by an upstream malignant, stromal, vascular or myeloid programme, or predominantly Treg independent? Second, does the tumour contain activated and therapeutically addressable tumour-adapted Tregs? Third, do these cells express a coherent suppressive programme rather than isolated FOXP3 or target expression? Fourth, are they positioned within anatomically and mechanistically plausible regulatory circuits? Fifth, does the tumour retain sufficient antigen-presentation capacity, recoverable effector immunity and intervention-specific machinery for disruption of the Treg circuit to restore antitumour activity?

The designation “Treg-niche positive” should be restricted to a provisional research classification in which these conditions converge and evidence supports functional Treg dependency rather than correlated infiltration. Treg-dependent tumours may be candidates for direct Treg depletion or reprogramming. Treg-reinforced tumours should be classified separately and may require mechanism-matched combination therapy. Tumours dominated by tumour-cell-intrinsic exclusion, irreversible antigen-presentation failure or absence of a recoverable effector compartment should be classified as Treg associated or predominantly Treg independent and should not be selected for isolated Treg-directed therapy. This operational stratification framework and its corresponding therapeutic implications are summarized in Figure 2C.

### A practical biomarker pipeline for Treg-niche stratification

The biomarker domains described above require integration into a clinically testable workflow. No individual marker, spatial metric or analytical platform is sufficient to establish Treg dependency. The proposed pipeline therefore separates pre-analytical qualification, causal assignment, targetability assessment, spatial confirmation and functional validation.

The first stage is pre-analytical qualification. Pretreatment tissue should include representative tumour, invasive-margin and stromal regions whenever available, with pathological annotation of viable tumour, necrosis, lymphoid aggregates and treatment-related changes. Fixation duration, cold-ischaemia time, section thickness, storage conditions and prior decalcification should be documented because these variables can affect epitope preservation, RNA quality, cell segmentation and spatial measurements. Formalin-fixed paraffin-embedded tissue should be prioritized for clinically deployable assays, whereas fresh or frozen specimens may support discovery-level transcriptomic and functional analyses.

The second stage should determine whether the dominant resistance architecture is plausibly Treg dependent before target-positive Tregs are used to guide treatment. Candidate features of Treg-independent or Treg-reinforced resistance include tumour-cell WNT–β-catenin activation, B2M or MHC class I loss, defective IFN-γ–JAK signalling, deficient cDC1 recruitment, lymphocyte depletion, dominant fibroblast or myeloid exclusion and absence of a recoverable CD8^+^ compartment (Spranger et al., 2015; Zaretsky et al., 2016; Shin et al., 2017). These measurements should determine whether Tregs are plausible primary effectors, downstream amplifiers or correlated bystanders. A tumour should not advance to an isolated Treg-directed strategy solely because it contains abundant, activated or spatially organized Tregs.

The third stage should establish whether the tumour contains an addressable regulatory population. Reduced multiplex immunofluorescence or serial immunohistochemistry should assess pan-cytokeratin, CD3, CD4, CD8, FOXP3, CD25, CCR8, CTLA-4 and Ki-67. Therapy-specific modules may incorporate TIGIT, ICOS, dendritic-cell, macrophage, stromal or vascular markers and, for Fc-engineered antibodies, NK-cell and Fcγ-receptor markers. This stage should establish Treg identity, activation and target expression without assuming that target presence establishes functional dependency.

The fourth stage should determine whether activated, target-positive Tregs occupy mechanistically plausible suppressive circuits. Prespecified measurements should include compartment-specific Treg density, target-positive Treg frequency, CD8/Treg ratios, Treg–CD8 and Treg–DC distances or contact frequencies, enrichment at tumour–stroma boundaries and proximity between target-positive Tregs and FcγR-expressing effector cells. These measurements should be evaluated against the spatial distribution of tumour-intrinsic, stromal and myeloid resistance programmes. Region-selection procedures, cell-classification rules, neighbourhood definitions and distance thresholds should be prespecified before validation because these analytical decisions can materially affect spatial results (Barua et al., 2018; Jackson et al., 2020; Schapiro et al., 2022b).

The fifth stage should distinguish functional Treg dependency from colocalization or shared recruitment. Transcriptomic profiling should determine whether identified Tregs express a tumour-adapted suppressive programme and whether competing tumour-intrinsic, antigen-presentation, IFN-γ, TGF-β, myeloid or stromal programmes dominate the same tissue. During discovery and early clinical development, candidate dependencies should be evaluated orthogonally using flow cytometry, tumour explants, organotypic slices, suppression assays, selective perturbation or early on-treatment biopsies (De Simone et al., 2016; Glasner et al., 2023).

Patient classification should be assigned according to causal category rather than Treg presence alone. A provisional Treg-dependent classification should require convergent evidence of therapeutically addressable Tregs, a coherent suppressive programme, localization within a plausible regulatory circuit, residual antigen-presentation and effector competence and compatibility between the proposed intervention and the machinery required for its activity. A Treg-reinforced classification should be used when an upstream malignant, stromal, vascular or myeloid programme remains active but Tregs make a demonstrable downstream contribution; these tumours should be considered for combination rather than isolated Treg-directed therapy. Tumours containing target-positive Tregs but lacking evidence of functional dependency or recoverable immunity should remain classified as Treg associated or predominantly Treg independent.

Candidate thresholds and exclusion rules should be defined separately for each tumour type, therapeutic mechanism and intended context of use, established in training cohorts and locked before independent validation. Validation must distinguish analytical performance, biological validity, clinical validity and clinical utility. Predictive validation should demonstrate that treatment benefit differs according to the biomarker-defined causal category rather than showing prognostic association alone. Baseline and on-treatment specimens should additionally determine whether target engagement, Treg depletion or functional reprogramming restores local immune activity.

Clinical deployment will ultimately require reduction of high-dimensional discoveries to an FFPE-compatible assay with limited tissue requirements, acceptable turnaround time and prespecified decision rules. Until analytical reproducibility, prospective predictive validity and clinical utility are demonstrated, this pipeline should be regarded as a framework for biomarker development rather than a validated clinical diagnostic algorithm.

## Safety and future directions for spatially guided Treg immunotherapy

The central challenge for Treg-directed cancer immunotherapy is that tumour tolerance and systemic self-tolerance rely on the same biological machinery. FOXP3^+^ Tregs are indispensable for immune homeostasis, tissue protection and prevention of autoimmunity; therefore, therapeutic disruption of this compartment must be restricted as much as possible to malignant tissue (Fontenot et al., 2003). The objective is not to abolish immune tolerance globally, but to induce a tumour-restricted collapse of the regulatory circuits that protect malignant tissue while preserving the Treg networks that protect healthy organs. This safety principle was recognized early in cancer immunology: Treg depletion can restore antitumour immunity, but nonspecific removal of regulatory cells risks autoimmune complications, highlighting the need for a therapeutic window that separates tumour-local immune restoration from systemic loss of self-tolerance (Beyer and Schultze, 2006).

The clinical history of immune checkpoint blockade provides a cautionary framework. CTLA-4 and PD-1 pathway blockade can produce durable tumour control, but they can also induce immune-related adverse events across multiple organs, including colitis, dermatitis, endocrinopathies, hepatitis and less frequent pulmonary, cardiac, neurologic, renal and haematologic toxicities (Hodi et al., 2010; Postow et al., 2018). Anti-CTLA-4 therapy is especially instructive because it acts early in T-cell activation, affects the regulatory compartment and is generally associated with broader immune toxicity than PD-1-axis blockade. These lessons should shape anti-Treg therapy: efficacy must be judged against the risk of breaking physiological tolerance.

Systemic versus intratumoral targeting is the defining safety axis. Systemic Treg depletion can amplify antitumour immunity, but it can also release autoreactive, commensal-reactive and tissue-reactive lymphocytes. Tumour-selective depletion or reprogramming aims to spare circulating and tissue-protective Tregs while disrupting tumour-adapted suppressive states. This therapeutic window will depend on target specificity, antibody format, Fcγ receptor engagement, tissue biodistribution, dose intensity, schedule and reversibility. Preclinical studies of Fc-dependent anti-CTLA-4, Fc-optimized anti-CD25 and CCR8-targeted antibodies show that antitumour efficacy can be driven by intratumoral Treg depletion and improved effector-to-regulatory T-cell balance (Simpson et al., 2013; Arce Vargas et al., 2017; Van Damme et al., 2021). However, human translation requires caution, because anti-CTLA-4 immunotherapy does not necessarily deplete FOXP3^+^ Tregs in human tumours to the same extent observed in some mouse models (Sharma et al., 2019). A tumour-enriched target is therefore not automatically safe or effective if treatment also affects activated Tregs in inflamed normal tissues, fails to engage the appropriate FcγR-expressing effector cells, or produces prolonged systemic regulatory collapse.

Dose, timing and sequencing should be treated as safety variables. Short, pulsed Treg disruption may reopen a window of antigen presentation and effector-cell expansion, whereas sustained depletion may increase immune-related toxicity. Sequencing may also alter risk: Treg disruption before, during or after antigen-releasing therapies could generate different balances between tumour immunity and systemic inflammation. The safest regimens will probably produce transient tumour-local immune permissiveness while allowing recovery or preservation of regulatory networks in healthy tissues.

Clinical trials should include pharmacodynamic evidence of selectivity. It will not be enough to show that a drug reduces FOXP3^+^ cells or increases CD8^+^ infiltration in bulk tumour samples. Trials should determine whether tumour Tregs were selectively depleted or reprogrammed, whether peripheral and normal-tissue Tregs were preserved, whether effector cells entered previously suppressed tumour regions and whether early inflammatory signals predict benefit or toxicity. Safety monitoring should include predefined stopping rules, early detection of gastrointestinal, cutaneous, hepatic and endocrine inflammation, and follow-up long enough to capture delayed immune toxicity.

The future of the field should be built around longitudinal human tissue evidence. Baseline biopsies can identify whether a tumour contains an actionable Treg niche, but on-treatment and post-treatment biopsies are needed to determine whether therapy remodels that niche. This principle is supported by human anti-PD-1 studies showing that spatially localized pre-existing CD8^+^ T cells at the invasive margin, together with on-treatment immune reinvigoration, can distinguish responding from non-responding tumours (Tumeh et al., 2014). For Treg-directed therapy, the critical questions are dynamic: did the suppressive circuit collapse locally; did dendritic-cell and myeloid states become more permissive; did CD8^+^ T cells enter tumour regions previously excluded; did stromal or vascular barriers change; and did these tissue changes correlate with response, resistance or toxicity?

Spatial transcriptomics and multiplex imaging should therefore become clinical pharmacodynamic tools. These methods can determine whether anti-Treg therapy produces a tumour-restricted collapse of suppressive architecture or only changes bulk immune composition. They can also identify whether related regulatory circuits are disrupted in adjacent normal tissue, providing early warning of toxicity. Spatially resolved single-cell pathology has shown that multiplex imaging can identify tumour, stromal and immune phenotypes, organize them into multicellular communities, quantify cell–cell interactions and define tissue architectures associated with clinical outcome (Jackson et al., 2020). Spatial readouts should therefore move from exploratory endpoints to integrated trial components that link mechanism, response and safety.

*Ex vivo* human models will be essential for prioritizing interventions before exposing patients to unnecessary risk. Tumour explants, organotypic slices and patient-derived organoid systems that retain immune and stromal components can test whether a given tumour is susceptible to Treg depletion, Treg reprogramming or neither. Organotypic tumour spheroid platforms have already shown that *ex vivo* systems can profile response to PD-1 blockade while preserving elements of the tumour microenvironment, supporting their use as translational tools for immunotherapy development (Jenkins et al., 2018). These systems can support short-term testing of antibody formats, Fc-dependent depletion and immune restoration in patient-specific contexts while preserving aspects of tissue architecture lost in dissociated assays.

Humanized models and improved preclinical platforms will remain important, but they should be interpreted cautiously. Mouse models have defined key principles of Treg biology and tumour immunity, yet Fcγ receptor biology, Treg marker specificity, tumour architecture and immune-related adverse events do not always translate directly to humans. The most informative development path will combine humanized models, autologous tumour–immune systems, organotypic platforms and longitudinal clinical biopsies.

Future anti-Treg trials should be biomarker-driven from the outset. Patients should not be enrolled simply because FOXP3^+^ cells are present or because a tumour type is historically immunogenic. Trial design should distinguish tumours that require selective Treg depletion from those that require functional reprogramming of the suppressive niche. Some tumours may be dominated by targetable tumour-enriched Tregs; others may be governed by stromal, myeloid, metabolic or vascular programmes that stabilize Treg-mediated tolerance. Still others may lack sufficient effector immune competence for anti-Treg therapy to be meaningful.

The long-term opportunity is spatially guided combination immunotherapy. In this framework, combinations are selected according to the architecture of immune failure rather than by empirical addition of agents. A tumour with Tregs constraining dendritic-cell restimulation, a tumour with Tregs embedded in stromal exclusion zones and a tumour with Tregs suppressing an inflamed CD8^+^ tumour bed may require different interventions. This principle aligns with the broader transition toward next-generation combination immunotherapy, in which checkpoint blockade, vaccines, adoptive cell therapies, cytokine-based agents, oncolytic viruses, synthetic immunomodulators, microbiome modulation, chronotherapy, advanced delivery platforms and AI-assisted modelling are increasingly integrated through biomarker-guided and context-specific strategies rather than uniform treatment escalation (Kyriakidis et al., 2025). This logic is especially important in metastatic disease, where organ-specific Treg programmes in sites such as liver, lung, bone, skin, brain, adrenal gland and lymph nodes may impose distinct thresholds for immune activation, tolerance and response to checkpoint blockade (Huppert et al., 2022). Spatial guidance could transform anti-Treg therapy from broad immune activation into precision remodelling of pathological tolerance. The goal is not to abolish immune tolerance, but to induce a tumour-restricted collapse of pathological tolerance while preserving systemic immune homeostasis.

## Discussion

The central implication of this review is that Treg-mediated tumour tolerance is not an intrinsic property of FOXP3^+^ cells considered in isolation, but an emergent property of tissue architecture. Tumour Tregs become therapeutically relevant when their cellular state, anatomical position and interactions with malignant, stromal, vascular, myeloid and effector compartments generate a suppressive circuit on which the tumour functionally depends. This distinction separates Treg abundance from Treg dependency and spatial association from therapeutic actionability. It also explains why apparently similar Treg-rich tumours may respond differently to regulatory-cell perturbation: in some, Tregs execute or stabilize the dominant suppressive mechanism; in others, they accompany immune exclusion established primarily by tumour-intrinsic or stromal programmes (Glasner et al., 2023).

The major unresolved challenge is therefore causal assignment. Current spatial technologies can identify cellular neighbourhoods, compartmentalization and candidate molecular interactions, but static proximity cannot establish which population initiates or maintains immune failure. The next phase of the field must combine spatial measurement with selective perturbation and temporal observation. Baseline maps should be linked to early on-treatment and progression specimens to determine whether candidate regulatory circuits collapse, relocate, recruit compensatory populations or persist despite target engagement. Greater use of paired human biopsies, tumour explants, organotypic tissue systems and autologous tumour–immune models will be essential because Fc-receptor biology, stromal architecture, tissue-specific Treg states and immune toxicity are not fully reproduced by conventional mouse models.

A decisive translational constraint is whether the tumour retains the immune machinery required to convert regulatory-circuit disruption into tumour rejection. Intervention will be insufficient when antigen presentation is irreversibly impaired, dendritic-cell recruitment is absent or the tumour lacks a recoverable tumour-reactive CD8^+^ compartment. Conversely, target-positive Tregs may be actionable when they coexist with competent antigen-presenting cells and TCF1^+^ stem-like or progenitor-exhausted CD8^+^ populations capable of sustaining effector-cell replenishment (Siddiqui et al., 2019; Miller et al., 2019). Future studies should therefore evaluate regulatory dependency and effector recoverability together rather than treating Treg markers as autonomous predictors of therapeutic benefit.

Clinical translation will ultimately require a shift from target-centred to architecture-centred trials. Target expression may establish biological addressability, but it should not define eligibility by itself. Trials must determine whether treatment remodels the intended intratumoral circuit, preserves systemic and tissue-resident Treg functions, restores spatial access and effector activity, and produces clinical benefit beyond conventional biomarkers. Recent CCR8 and LAG-3 studies illustrate that target expression, pharmacodynamic remodelling and clinical efficacy represent distinct evidentiary stages (Van Damme et al., 2021; Long et al., 2025; Wang et al., 2026). The objective is therefore not maximal Treg depletion, but tumour-restricted disruption of pathological tolerance. Achieving this will require prospective validation of tumour-type-specific regulatory dependencies and rational combinations selected according to the causal architecture of immune failure rather than empirical treatment escalation.
